# Repopulating Microglia Suppress Peripheral Immune Cell Infiltration to Promote Poststroke Recovery

**DOI:** 10.1111/cns.70565

**Published:** 2025-09-10

**Authors:** Ligen Shi, Lingxiao Lu, Jun Hu, Jiarui Chen, Qia Zhang, Ziyang Jin, Zhen Wang, Zhe Zheng, Jianmin Zhang

**Affiliations:** ^1^ Department of Neurosurgery Second Affiliated Hospital, School of Medicine, Zhejiang University Hangzhou Zhejiang China; ^2^ Research Center for Life Science and Human Health, Binjiang Institute of Zhejiang University Hangzhou China; ^3^ Clinical Research Center for Neurological Diseases of Zhejiang Province Hangzhou China

**Keywords:** immunomodulation, neuroinflammation, PLX5622, replenished microglia

## Abstract

**Aims:**

Sustained neuroinflammation following ischemic stroke impedes post‐injury tissue repairment and neurological functional recovery. Developing innovative therapeutic strategies that simultaneously suppress detrimental inflammatory cascades and facilitate neurorestorative processes is critical for improving long‐term rehabilitation outcomes.

**Methods:**

We employed a microglia depletion‐repopulation paradigm by administering PLX5622 for 7 days post‐ischemia; followed by a 7‐day withdrawal period to allow microglia repopulation. Single‐cell transcriptomics, behavioral testing, cytokine arrays, flow cytometry, and immunofluorescence were used to assess the effects of microglia repopulation and delineate the transition of reshaped immune microenvironment.

**Results:**

PLX5622 administration reshaped the poststroke immune microenvironment, promoting neurofunctional recovery. Repopulated microglia adopted a homeostatic phenotype, increasing homeostatic states by ~14.36% and reducing pro‐inflammatory states by ~20.17%. This reshaped environment suppressed T cell exhaustion, limited neutrophil terminal differentiation, and promoted a phagocytic macrophage phenotype. Furthermore, we identified that these transitions in infiltrating immune cells may be driven by reduced chemokine production, enhanced blood–brain barrier (BBB) integrity, and transcriptional reprogramming.

**Conclusion:**

Transient microglial depletion and repopulation via PLX5622 during the acute phase post stroke facilitate the recovery of neurological function. This immunomodulatory strategy offers a promising and clinically translationally relevant approach to enhance functional recovery following ischemic brain injury.

## Graphical abstract

PLX5622 treatment during the acute phase of tMCAO reshapes the immune microenvironment. The repopulated microglia adopt a homeostatic phenotype, contributing to improved blood–brain barrier integrity and inhibiting the recruitment of peripheral immune cells. The infiltrating immune cells undergo phenotypic shifts that favor neurorepair and functional recovery.

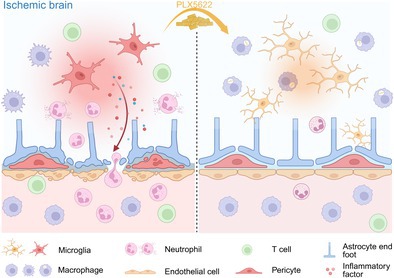

## 1 Introduction

Stroke is one of the leading causes of death and disability worldwide [1]. With advances in treatment techniques, an increasing number of patients achieve vascular reperfusion through thrombolysis and mechanical thrombectomy in the early stages. However, vascular reperfusion fails to fully prevent persist inflammatory responses and neuronal loss, resulting in more than 50% of patients experiencing moderate to severe functional disabilities [2, 3]. Still, an effective therapy targeting secondary injury remains to be established. Numerous immune cells infiltrate into the infarct region and persist for an extended period [4, 5], contributing to the sustained presence of inflammation [6]. Thus, immunomodulation appears to be a promising approach [7, 8]. Immune cells initially exert pro‐inflammatory effects during the acute phase of stroke but transition to reparative roles in the chronic phase, aiding recovery by producing neurotrophic factors and synapse‐modulating molecules [7]. Therefore, developing therapeutics that suppress acute inflammation while preserving subsequent reparative processes holds significant therapeutic potential [9].

Microglia, the brain's resident immune cells, exert biphasic roles throughout stroke progression. During the acute phase, they initiate the inflammatory response and promote the recruitment of peripheral immune cells [10]. Subsequently, they transition toward a reparative role [11], fostering an anti‐inflammatory and pro‐recovery microenvironment within the brain. However, sustained microglial overactivation can hinder the transition of the immune microenvironment toward a reparative state following ischemic stroke [12]. Therefore, timely modulation of microglia to suppress pro‐inflammatory activation while preserving their pro‐repair potential represents a key strategy for enhancing tissue repair following brain injury [13, 14].

Colony stimulating factor 1 receptor (CSF1R) inhibitors, such as PLX5622, have been demonstrated to selectively suppress microglial proliferation, effectively depleting microglia from the mouse brain [15]. Hence, an increasing number of studies have explored microglia depopulation and repopulation strategies, aiming to regulate dysregulated microglia activity in various neurological diseases. Recent studies have shown that prolonged microglia depletion does not improve neurofunctional recovery and even aggravates damage, as observed in stroke [16] and spinal cord injury (SCI) [17]. In contrast, microglia repopulation has been found to facilitate neurofunctional recovery in various conditions involving brain inflammation and neurodegeneration, including stroke [18], traumatic brain injury (TBI) [19], AD [20], intracerebral hemorrhage (ICH) [21] and SCI [22]. These findings underscore the indispensable role of microglia in maintaining a homeostatic microenvironment and highlight microglia repopulation as a promising therapeutic strategy. However, most studies induce microglia repopulation by depleting microglia prior to injury onset, and there is a lack of comprehensive understanding regarding how repopulated microglia reshape the brain immune microenvironment and undergo phenotypic transitions after stroke. Moreover, since brain injuries in clinical settings are typically unpredictable, pre‐injury microglia depletion is impractical. These limitations highlight the urgent need to explore post‐injury depletion strategies and to elucidate the mechanisms by which repopulated microglia modulate the brain's immune landscape. Therefore, acute stage depletion of microglia to avoid inflammatory environment, followed by the repopulation of reshaped microglia, may offer a promising immune regulatory strategy to enhance long‐term neurological recovery after stroke and hold greater potential for clinical translation.

In this study, we administered PLX5622 during the early phase to depopulate microglia and prevent their overactivation; then we withdrew the treatment in the subacute phase to permit microglia repopulation, allowing them to exert protective effects. We found those mice treated with PLX5622 during the early phase after ischemic stroke had significantly enhanced neurological recovery. Our findings revealed that microglia replenishment resulted in a 14.36% increase in homeostatic states and a 20.17% reduction in pro‐inflammatory phenotypes, accompanied by diminished infiltration of peripheral inflammatory immune cells. Together, these changes contributed to the establishment of a more homeostatic and pro‐repair microenvironment during the chronic stage. We further identified that microglia repopulation suppressed chemokine production, downregulated adhesion molecule expression on endothelial cells, and enhanced BBB integrity, collectively limiting immune cell recruitment. Furthermore, the reshaped microenvironment reprograms infiltrating immune cells, thereby preventing their transition into pro‐inflammatory phenotypes. These findings propose a novel immunomodulation strategy that reshapes the poststroke immune microenvironment toward a more homeostatic state, thereby creating favorable conditions for neurofunctional recovery and offering greater potential for clinical translation.

## 2 Result

### 2.1 PLX5622 Therapy Leads to Resolution of Inflammation in Chronic Phase of Ischemic Stroke

**FIGURE 1:**
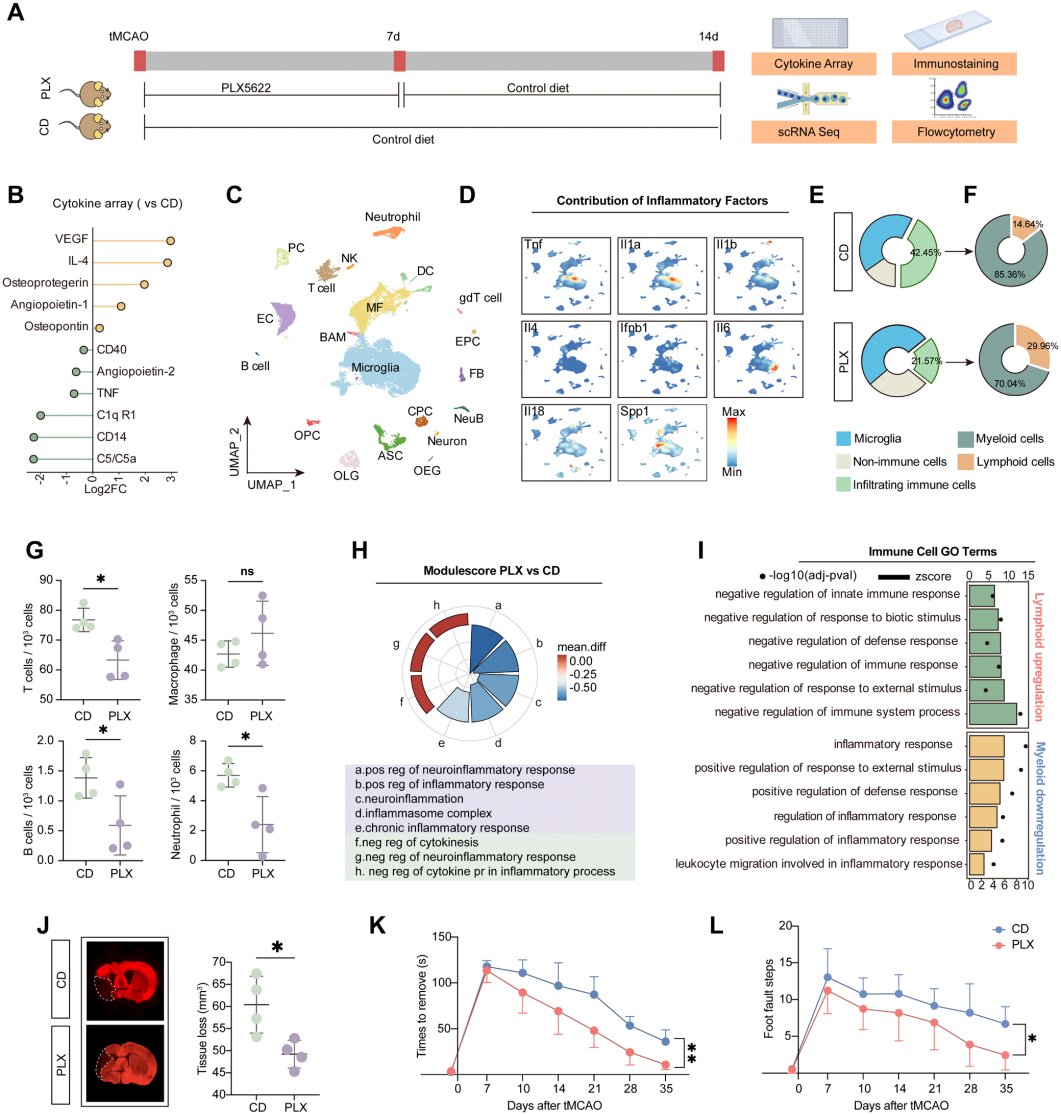
PLX5622 therapy leads to resolution of inflammation in the chronic phase of ischemic stroke. (A) Experimental scheme illustrating v the PLX5622 diet of C57/BL6 mice after tMCAO. (B) Quantification of cytokines array in PLX group or CD group (*n* = 3 for each group). (C) UMAP projection plot showing cell clusters from the ipsilateral brain at 14 dpi. (D) Density plots in the UMAP space showing the expression of inflammatory factors. Scale bar represents densities based on Kerenel density estimation of gene expression. (E) The chart plot represents the proportion of cells from the ipsilateral brain at 14 dpi. (F) The chart detailed the composition of immune cells shown in the left chart. (G) Dot plots showing the number of immune cells (among 1000 cells in ipsilateral brain) with or without PLX5622 administration. *n* = 4 for each group. ns = not significant. *p < 0.05. Student's *t* test, two‐sided (T cells: *p* = 0.0119, B cells: *p* = 0.0379, Neutrophil: *p* = 0.0177, Macrophage: *p* = 0.2771). (H) Showing are radial plots indicating the differences of scores about inflammation associated gene sets. Bar height represents the difference in medians between PLX group and CD group. Color indicates the difference in medians between PLX group and CD group. (I) A bar plot showing the activated (*Z* > 2) GO terms involved which upregulated in PLX group lymphoid cells, downregulated in PLX group myeloid cells. Bar height represents the value of −log10 (adj‐pval). (J) Quantification of tissue loss at 14 dpi in MAP2‐stained (red) coronal sections. *n* = 4 per group. Student's *t* test, two‐side (*p* = 0.0204). (K) Adhesive removal test. *n* = 7 for CD group, *n* = 8 for PLX group. 2‐way ANOVA repeated measurement (*p* = 0.0023). (L) Foot fault test. *n* = 7 for CD group, *n* = 8 for PLX group. 2‐way ANOVA repeated measurement (*p* = 0.0289).

In this study, we aim to elucidate how microglia depletion and subsequent repopulation influence functional recovery following stroke. A transient middle cerebral artery occlusion (tMCAO) model was established by subjecting mice to 60 min of occlusion. Following surgery, mice in the PLX5622 treated (PLX) group received a diet containing the CSF1R inhibitor PLX5622 [23] for 7 days, while control diet (CD) mice received a standard diet. Both groups were subsequently maintained on the control diet for an additional 7 days. Flow cytometry, immunostaining, cytokine analysis, and single‐cell RNA sequencing were employed to evaluate the effects of PLX5622 treatment (Figure 1A).

To confirm effective microglia depletion by PLX562, flow cytometry at 7 days post tMCAO was performed, revealing an approximately 70% reduction in microglia in the PLX group (Figure S1A). Then, to evaluate changes in the ipsilateral microenvironment, inflammatory cytokines were quantified using a mouse cytokine array at 14 days post tMCAO. In the PLX group, pro‐inflammatory factors such as CD14, CD40, TNF, and complement‐associated molecules were reduced, while anti‐inflammatory cytokines, including IL‐4 and Osteopontin (*Spp1*), were elevated (Figure 1B), indicating an attenuated inflammatory environment after microglia repopulation.

To further characterize changes in the immune landscape, single‐cell RNA sequencing was performed on cells isolated from the ischemic hemisphere at 14 days post‐injury (dpi) in both the PLX and CD groups (Figure 1C, Figure S1C). A total of 23,782 cells passed quality control and underwent downstream analyses. Unsupervised clustering identified 20 distinct cell populations, including Microglia, monocytes, and monocyte‐derived macrophage (MF), border‐associated macrophages (BAM), dendritic cells (DC), Neutrophil, natural killer cells (NK cell), T cells, gamma delta T cells (gdT cell), B cell, Neuron, neuroblasts (NeuB), astrocytes (ASC), oligodendrocytes (OLG), oligodendrocyte progenitor cells (OPC), endothelial cells (EC), pericytes (PC), fibroblast‐like cells (FB), ependymocytes (EPC), choroid plexus epithelial cells (CPC), and olfactory ensheathing glial cells (OEG) (Figure 1C, Figure S1C). Analysis of the distribution of inflammatory cytokines revealed that microglia and infiltrating immune cells were the primary sources of both pro‐inflammatory and anti‐inflammatory mediators (Figure 1D). Furthermore, we found that, compared to the CD group, the cellular composition in the PLX group was markedly altered (Figure S1D), especially these immune cells (Figure 1E). Overall, microglia accounted for a higher proportion, while infiltrating immune cells had a reduced proportion in the PLX group (Figure 1E). Further analysis of the infiltrating immune compartment revealed a reduction in myeloid cells and an increased proportion of lymphoid cells in the PLX group (Figure 1F).

To further assess the role of microglia repopulation in modulating peripheral immune cell infiltration, flow cytometry was performed at 14 days post tMCAO. We found that the PLX group exhibited reduced infiltration of B cells and T cells, while macrophage proportions remained unchanged (Figure 1G, Figure S1F), suggesting that microglia repopulation suppresses peripheral immune cell recruitment, consistent with previous findings [18]. To determine whether microglia depletion or repopulation accounts for the reduced peripheral immune cell infiltration, flow cytometry was performed at 5 days post stroke. The analysis revealed an increased number of infiltrating macrophages, while other immune cell types remained unchanged (Figure S1B). These findings suggest that it is the repopulated microglia, rather than depletion itself, that contributes to the suppression of peripheral immune cell recruitment.

Then, AddModuleScore was applied to evaluate differences in microglia inflammatory functions between the two groups (Figure 1H). We found that microglia in the PLX group exhibited a distinct functional shift, characterized by decreased pro‐inflammatory scores and increased anti‐inflammatory scores (Figure 1H). To characterize changes in infiltrating immune cells, differentially expressed genes (DEGs) between the PLX and CD groups were analyzed through Gene Ontology (GO) enrichment (Table S4, Figure S1E). We found that lymphoid cells from the PLX group were enriched in functions related to the negative regulation of inflammatory responses, including negative regulation of immune effector processes and negative regulation of TNF production, while myeloid cells showed downregulation of pathways promoting inflammation (Figure 1I). Collectively, these results suggest that early‐phase PLX5622 treatment after stroke reprograms immune cells and suppresses excessive inflammatory responses.

To confirm the protective effect of PLX5622 treatment, MAP2 immunostaining was applied at 14 days. A significant reduction in tissue loss was observed in the PLX group compared to the CD group (Figure 1J). Adhesive removal and foot fault tests were conducted to evaluate long‐term neurological function. Consistent with reduced infarct volume, mice with transient microglial depletion‐repopulation exhibited improved behavioral performance (Figure 1K,L). Collectively, these data indicate that microglial depletion‐repopulation in the early phase of stroke enhances neurological recovery.

In summary, our results indicate that acute‐phase PLX5622 treatment shifts the immune microenvironment in the infarcted brain from a pro‐inflammatory to an anti‐inflammatory state; thereby promoting neurological recovery.

### 2.2 The Effect of PLX5622 on Peripheral Immune Cells

Although PLX5622 is primarily used to deplete microglia, recent studies have demonstrated its off‐target effects [24]. To further investigate the impact of PLX5622 on peripheral immune cells, we performed flow cytometry on blood samples at 5 days post tMCAO (Figure S2A). In our study, no significant changes were observed in the composition of major peripheral immune cells, including B cells, T cells, and NK cells (Figure S2B). However, a reduction in circulating monocytes was detected (Figure S2C), suggesting that PLX5622 does have an off‐target effect specifically on peripheral monocytes without impacting other major immune cell populations.

To comprehensively evaluate functional alterations in peripheral immune cells following PLX5622 administration, single‐cell RNA sequencing of peripheral blood was performed at 5 days post tMCAO in both PLX and CD groups (Figure S2D). After quality control and unsupervised clustering analysis, nine distinct cell types were identified, including neutrophil, B cells, T cells, NK cells, Ly6C+ monocyte, Megakaryocyte, Ly6C‐ monocyte, Basophil, and DCs (Table S4, Figure S2D,E). Principal component analysis (PCA) showed spatial proximity of major immune cell types (Neutrophil, B cells, T cells, NK cells, Ly6C+ monocyte and DCs) between CD and PLX groups (Figure S2F), suggesting that the major immune cell populations in both groups share highly similar gene expression profiles. Additionally, AddModuleScore analysis using inflammation‐associated gene sets showed similar scores between the two groups across major peripheral immune cell types (Figure S2G), indicating a comparable inflammatory function between the two groups.

Overall, we found that PLX5622 treatment exerts off‐target effects primarily on peripheral monocytes, while its impacts on the composition and function of other peripheral immune cells remain limited.

### 2.3 Replenished Microglia Underwent Phenotype and Function Transformation

Microglia, the resident macrophage of the brain, play a pivotal yet dual role in both initiating and resolving inflammatory responses following tMCAO. Leveraging single cell data, we mapped the dataset from the PLX group onto the cluster identities of the CD group (Figure S3A), revealing that replenished microglia exhibited phenotypic similarities to microglia rather than infiltrating macrophages in the CD group (Figure S3G). Moreover, Sall1 and Sall3, two genes specifically expressed by yolk sac‐derived microglia [25, 26], are expressed in original microglia and repopulated microglia but not in macrophages (Figure S3H). These data suggest that repopulated microglia are mainly derived from residual microglia.

**FIGURE 2:**
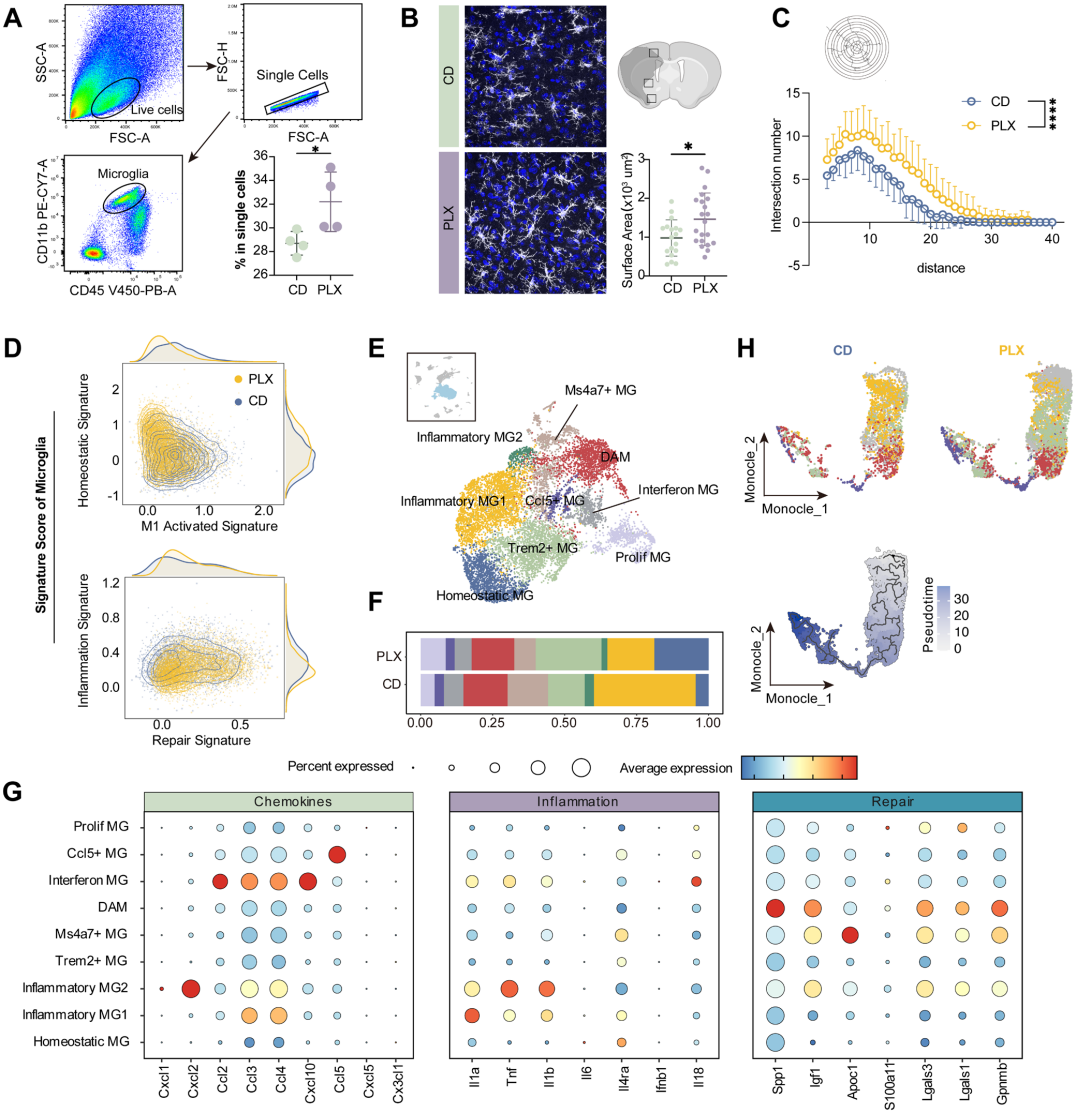
Replenished microglia underwent phenotype and function transformation. (A) Gating strategy for CD45^medium^CD11b^+^ microglia. Representative flow cytometry plot of microglia derived from the brain of tMCAO mice (left). Quantitative comparison of proportion of CD45^medium^CD11b^+^ microglia in ipsilateral brain at 14 dpi among mice with control diet or treated with PLX5622. *n* = 4 for each group. *p < 0.05, Student's *t* test (*p* = 0.0411) (low right). (B) Representative plot depicting the phenotypes of microglia in peri‐infract area (left), Cell surface area (low right) was quantified. *n* = 17 for CD group, *n* = 20 for PLX group. *p < 0.05, Student's *t* test (*p* = 0.0125). (C) Morphology statistics of microglia in the ipsilateral brain at 14 dpi by Sholl analysis; *n* = 22 for each group; mixed‐effects model (*p* < 0.0001). (D) Scatter density plots showing microglial signature scores under each group. Distribution of microglia along the homeostatic signature and M1‐activated signature axes (top). Distribution of microglia along the inflammation signature and repair signature axes (bottom). Yellow points represent PLX group, blue points correspond to the CD group. Density curves along the axes illustrate the overall distribution of microglial populations. (E) UMAP projection of microglia. (F) Stacked bar plot showing cell clusters from microglia at 14 dpi. *n* = 2 biological replicates for each group. (G) Expression of representative signature genes across microglia subpopulations. (H) Pseudotime trajectory analysis of microglia in the ischemic brain at 14 dpi using Monocle3. UMAP projection showing the distribution of microglial subpopulations in the PLX and CD groups (top). UMAP projection illustrating the pseudotime trajectory of microglia, highlighting their differentiation pathways (bottom).

Then, to quantify changes in microglial proportions, flow cytometry was performed at 14 days post stroke. Consistent with single‐cell RNA sequencing results (Figure S1D), a higher proportion of microglia was observed in the PLX group (Figure 2A). Immunostaining of IBA1 in peri‐infarct regions revealed an increased microglia surface area (Figure 2B) and a higher proportion of TMEM119^+^IBA1^+^ microglia (Figure S3C). Sholl analysis further demonstrated enhanced process complexity in the PLX group (Figure 2C), suggesting that replenished microglia adopt a more homeostatic phenotype.

To compare the functional transitions of microglia after PLX5622 treatment, signature modules of homeostatic, M1‐activated, repair, and inflammatory states were used for AddModuleScore analysis. Microglia from the PLX group exhibited higher scores in homeostatic and repair signatures, alongside lower scores in inflammatory and M1‐activated signatures (Figure 2D). These findings suggest that repopulated microglia exhibit drastic phenotypic alterations, prompting us for a further analysis of microglia characteristics. Reanalysis of microglial populations identified ten distinct subpopulations, including Homeostatic Microglia (Homeostatic MG, Tmem119), Inflammatory Microglia 1 (Inflammatory MG1, Il1a), Inflammatory Microglia 2 (Inflammatory MG2, Cxcl2), Trem2+ Microglia (Trem2+ MG, Trem2), Ms4a7+ Microglia (Ms4a7+ MG, Ms4a7), Disease‐Associated Microglia (DAM, Spp1), Interferon‐Associated Microglia (Interferon MG, Ifit1), Ccl5+ Microglia (Ccl5+ MG, Ccl5), and Proliferative Microglia (Prolif MG, Mki67) (Figure 2E, Table S4).

Among them, DAM exhibited higher expression of Spp1, Igf1, and Itgax (Figure S3D), consistent with previously defined DAM in earlier studies [27, 28], which exert a pro‐recovery function after tMCAO. Then, DEGs of DAM were mapped onto the dataset from Aymerie Silvin et al. [29]; consistent with their findings, these DAMs had a higher similarity to immature microglia (Figure S3F). In addition, Ms4a7+ MG had a higher expression of Lyz2, Cybb, Ms4a7, and Itga4 (Figure S3B), resembling previously characterized microglia‐like macrophages of peripheral origin [26, 30]. By mapping the DEGs of these Ms4a7+ MG to the Aymerie Silvin et al. dataset, we found that non‐resident myeloid cells had a higher score of these genes (Figure S3C), suggesting Ms4a7+ MG may originate from peripheral infiltrating macrophages that have acquired a microglia‐like phenotype, as recently reported [29].

Compared to the CD group, the PLX group exhibited a higher proportion of homeostatic MG and Trem2+ MG while a marked reduction in Inflammatory MG1, Inflammatory MG2, and Interferon MG (Figure 2F), which are the primary sources of inflammatory cytokines (Figure 2G), indicating that PLX5622 treatment influences the differentiation trajectory of microglia.

To construct the differentiation trajectory of microglia, pseudotime analysis using Monocle3 was conducted, revealing distinct differentiation trajectories between the two groups (Figure 2H). Microglia in the CD group primarily differentiated toward the Inflammatory MG1, whereas those in the PLX group followed a trajectory toward DAM, which subsequently expanded through self‐proliferation (Figure 2H). Notably, in the PLX group, Trem2+ MG emerged upstream in the transition from Homeostatic MG to DAM (Figure 2H), consistent with previous studies identifying Trem2 as a key regulator in the formation of DAM [27]. To confirm the relationship between Trem2 and DAM after tMCAO, we leveraged the dataset from Lidia Garcia‐Bonilla et al. [31] and found that Trem2 expression positively correlates with time post‐injury (Figure S3E,F), consistent with the gradually increased proportion of DAM reported in their study [31]. Thus, we speculate that Trem2+ MG may represent an intermediate state that subsequently differentiates into DAM, exerting protective functions following tMCAO. After PLX5622 treatment, a greater proportion of replenished microglia may adopt this Trem2+ phenotype, thereby contributing to enhanced functional recovery.

Collectively, PLX5622 treatment induces substantial changes in microglial morphology, abundance, and function. These transformations help suppress inflammation and establish a homeostatic immune environment, ultimately supporting neurofunctional recovery.

### 2.4 PLX5622 Treatment Reshape the Constitution and Function of Infiltrated Immune Cells

**FIGURE 3:**
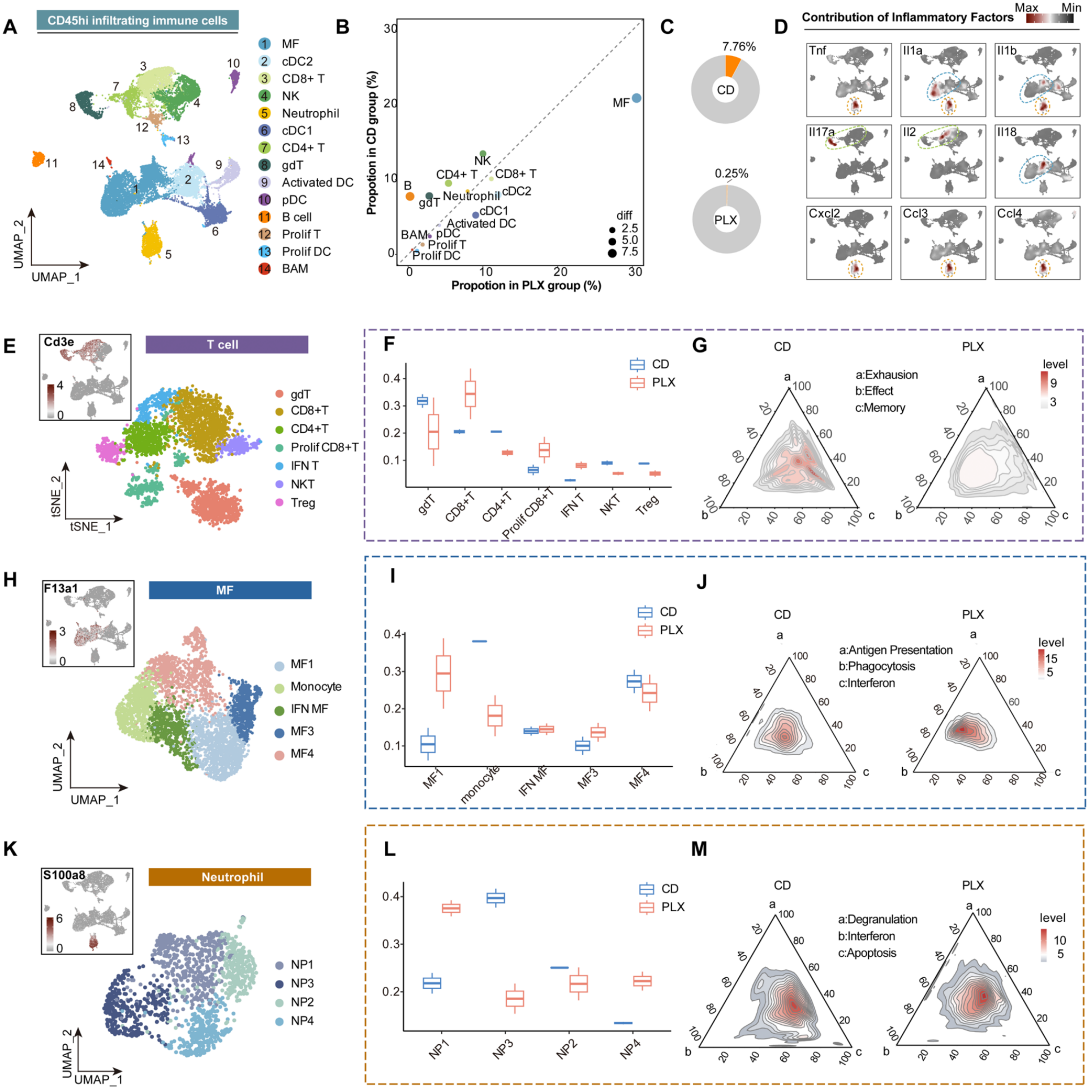
PLX5622 treatment reshape the constitution and function of infiltrated immune cells. (A) UMAP projection plot showing CD45^hi^ immune cells (remove microglia) from the ipsilateral brain at 14 dpi. (B) Dot plot illustrating the differences in the percentage of each immune cell type between the PLX group and the CD group. (C) Chart plot showing the proportion of B cells in CD group and PLX group. (D) Density plots in the UMAP space showing the expression of inflammatory factors and regulatory factors. Scale bar represents densities based on Kerenel density estimation of gene expression. (E) tSNE projection of T cells. (F) Box plot showing the proportion of different subpopulations of T cells in each group. (G) Ternary density plots comparing the distribution of T cells between the CD and PLX groups. The three axes represent different T cell states: A (Exhaustion), b (Effect), and c (Memory). Density contours indicate the relative abundance of cells within each category, with color intensity representing different levels of enrichment. (H) UMAP projection of MF. (I) Box plot showing the proportion of different subpopulations of MF in each group. (J) Ternary density plots comparing the distribution of MF between the CD and PLX groups. The three axes represent different MF states: A (Antigen Presentation), b (Phagocytosis), and c (Interferon‐associated). (K) UMAP projection of neutrophils. (L) Box plot showing the proportion of different subpopulations of neutrophils in each group. (M) Ternary density plots comparing the distribution of neutrophil between the CD and PLX groups. The three axes represent different neutrophil states: A (Degranulation), b (Interferon‐associated), and c (Apoptosis).

During the chronic phase following ischemic stroke, both microglia and infiltrating peripheral immune cells influence disease progression and functional recovery [7]. In our study, we found that PLX5622 treatment significantly altered the functions of these infiltrating immune cells (Figure 1H); however, due to their low abundance in the whole brain dataset, our understanding of their functional roles remains limited. To better understand the infiltrating immune cells after PLX5622 treatment, we established a tMCAO model. Mice in the PLX group received a PLX5622‐containing diet for 7 days, while the CD group received a control diet. Both groups were then maintained on the control diet for an additional 7 days. Subsequently, both groups of mice were sacrificed, and CD45^hi^ immune cells from the infracted hemisphere were isolated for single cell RNA sequencing (Figure 3A).

After quality control and removal of microglia, we identified 14 distinct immune cell populations: monocyte‐derived cells (MF), border‐associated macrophages (BAM), classical dendritic cells 1 (cDC1), classical dendritic cells 2 (cDC2), activated dendritic cells (Activated DC), plasmacytoid dendritic cells (pDC), natural killer cells (NK), CD8+ T cells (CD8+ T), CD4+ T cells (CD4+ T), gamma delta T cells (gdT), B cells (B), neutrophils, proliferative T cells (Prolif T), and proliferative dendritic cells (Prolif DC) (Table S4, Figure 3A). The application of PLX5622 induced notable compositional shifts within CD45^hi^ cells, characterized by an increased proportion of MF, CD8+ T cells, accompanied by reductions in CD4+ T cells and neutrophils (Figure 3B). Notably, the proportion of B cells drastically decreased from 7.76% to 0.25% in the PLX group (Figure 3C), which had recently been reported to contribute to microglia activation [32]. Therefore, their reduction in the PLX group may partially contribute to the transition of microglia toward a more homeostatic phenotype.

Building on our previous finding that PLX5622 treatment attenuates inflammation at 14 days post stroke (Figure 1), we aimed to identify the principal sources of inflammatory cytokines among CD45^hi^ cells and to characterize their functional and compositional heterogeneity following treatment. We identified neutrophil and MF as primary sources of inflammatory cytokines (Figure 3D), while T cells were the main sources of crucial modulators, such as *Il2* and *Il17a* (Figure 3D). Thus, to gain deeper insights of shifted inflammatory environment, neutrophil, T cells, and MF were extracted for further analysis.

We identified seven distinct subsets of T cells, including gamma‐delta T cells (gdT), CD8+ T cells, CD4+ T cells, proliferative CD8+ T cells, interferon‐associated T cells (IFN T), NKT cells, and regulatory T cells (Treg) (Figure 3E, Figure S4A, Table S4). Notably, in terms of T cell composition, the PLX group showed an increased proportion of CD8+ T cells and a decreased proportion of CD4+ T cells (Figure 3F). Flow cytometry confirmed an overall reduction in infiltrating T cells, encompassing both CD4+ and CD8+ subsets (Figure S4C); however, the CD4+ T cells/CD8+ T cells ratio showed a decreasing trend in the PLX group (Figure S4B), consistent with our single‐cell RNA results. Furthermore, we found that microglia highly expressed B2m, which encodes beta‐2‐microglobulin—a component of the MHC I molecules essential for CD8+ T cell persistence [33]—with notably high expression in the PLX group (Figure S4D). This suggests that microglia may support CD8+ T cell maintenance through enhanced TCR/pMHC interactions.

Then, to have a comprehensive understanding of the functional transformation of T cells, AddModuleScore was applied to assess exhaustion, effector, and memory signatures among T cells. Compared to the CD group, which showed greater enrichment in exhaustion and memory‐like T cell signatures, T cells in the PLX group predominantly exhibited effector‐like characteristics (Figure 3G).

For MF, they were clustered into five subpopulations: monocyte, MF1, MF3, MF4, and interferon‐associated macrophages (IFN MF) (Figure 3H, Figure S4E, Table S4). PLX5622 treatment marked altered macrophage composition, characterized by an increase in MF1 and MF3, and a reduction in MF4 and monocytes (Figure 3I). Module score analysis revealed that infiltrating macrophages in the PLX group exhibited a stronger phagocytosis‐related signature than those in the CD group (Figure 3J); among them, MF1 and MF3 were identified as the primary phagocytic subpopulations (Figure S4F). Based on MF1 marker Gpnmb (Figure S4E), immunostaining of MBP, GPNMB, and IBA1 was applied and confirmed that GPNMB^+^ macrophage exhibited enhanced phagocytic capacity (Figure S4G). Flow cytometry further validated the increased proportion of GPNMB+ macrophage in the PLX group (Figure S4H), suggesting that PLX5622 treatment promotes macrophage reprogramming toward a phagocytosis‐associated phenotype, potentially contributing to debris clearance and functional recovery.

Neutrophils were then subdivided into NP1, NP2, NP3, and NP4 (Figure 3K, Figure S4I, Table S4); among them, NP2 represents the early‐infiltrating neutrophil population, as they had a higher expression level of Sell and Cxcr2 (Figure S4K), consistent with previous reports of early‐infiltrating subtypes [34]. PLX5622 treatment significantly influenced neutrophil composition, with a marked decrease in NP3 (Figure 3L). Module analysis revealed that neutrophils in the PLX group showed reduced enrichment for apoptosis‐related signatures (Figure 3M), suggesting that PLX5622 treatment inhibited their transition to terminally differentiated states. Notably, NP3 exhibited the highest apoptosis‐related scores (Figure S4I) and served as a major source of inflammatory factors (Figure S4K), indicating NP3 plays a key role in sustaining the inflammatory response after stroke. Collectively, these findings indicate that early‐phase PLX5622 treatment alters the differentiation trajectories of infiltrating neutrophils, limits the emergence of pro‐inflammatory terminal subpopulations, and thereby fosters a more stable immune microenvironment.

In conclusion, our findings demonstrate that PLX5622 treatment alters both the composition and function of infiltrating immune cells. This shift toward anti‐inflammatory and pro‐repair phenotypes alleviates chronic inflammation and promotes recovery after ischemic stroke.

### 2.5 Replenished Microglia Inhibited the Recruitment of Peripheral Immune Cells

**FIGURE 4:**
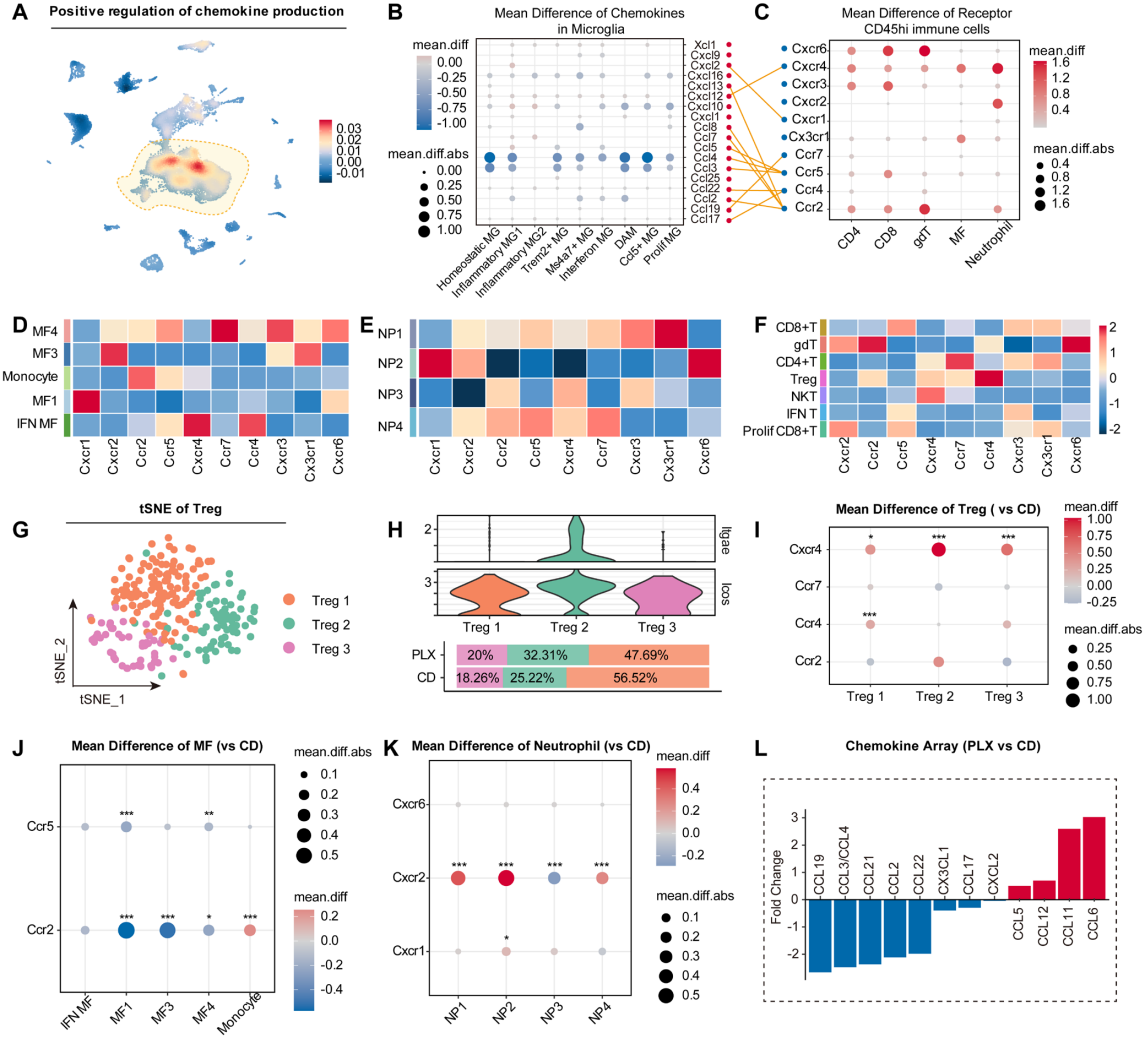
Replenished microglia inhibited the recruitment of peripheral immune cells. (A) Density plots in the UMAP space showing the Score of ‘positive regulation of chemokine production’. Scale bar represents densities based on Kerenel density estimation of gene expression. (B) Changes in the mean expression levels of key chemokines across microglial subpopulations between the CD group and the PLX group. (C) Changes in the mean expression levels of key chemokine receptors across main CD45^hi^ immune cells between the CD group and the PLX group. (D) Heatmap showing the changes in mean expression levels of key chemokine receptors across MF subpopulations. (E) Heatmap showing the changes in mean expression levels of key chemokine receptors across neutrophil subpopulations. (F) Heatmap showing the changes in mean expression levels of key chemokine receptors across T cell subpopulations. (G) tSNE projection of Tregs. (H) Violin plot comparing the expression levels of Itga3 and Icos across different clusters (top). Stacked bar charts represent the proportion of each cluster in the PLX group and CD group (bottom). (I, K) Changes in the mean expression levels of key chemokine receptor across Treg (I), MF (J) and neutrophil (K) subpopulations between PLX group and CD group. *p < 0.05, **p < 0.01, ***p < 0.005. Wilcox test. (L) Quantification of chemokines expression in PLX group compared to CD group (*n* = 3 for each group).

To elucidate why a reshaped immune microenvironment reduces peripheral immune cell infiltration following ischemic stroke, we initially focused on chemotaxis, a pivotal mechanism initiating peripheral immune cell migration. Firstly, module score analysis identified microglia as the primary source of chemokine production at 14 days post tMCAO (Figure 4A). Furthermore, chemokine‐associated gene sets analysis revealed significantly lower chemokine‐associated functional scores in microglia from the PLX group compared to the CD group (Figure S5A), suggesting that the reduced recruitment of peripheral immune cells may be attributed to decreased chemokine production by replenished microglia. For that, a more detailed comparison of microglia subpopulations was performed, revealing decreased expression of key chemokines—including Ccl2, Ccl3, Ccl4, and Cxcl16—across all subpopulations in the PLX group (Figure 4B). Together, microglia—the primary source of chemokines after stroke—revert to a more homeostatic state following replenishment, resulting in reduced chemokine production and limited infiltration of peripheral immune cells.

Unexpectedly, CD45^hi^ immune cells from the PLX group exhibited a significant upregulation of chemokine receptor expression (Figure 4C). Given that it might not accurately reflect receptor dynamics for those early‐infiltrating immune cell subpopulations, we conducted a more refined analysis. Firstly, we compared the expression level of various chemokine receptors across subpopulations of MF, neutrophils, and T cells (Figure 4D–F). Ccr2 and Ccr5 were identified as the predominant receptors on monocytes, the initially infiltrating population of MF (Figure 4D). For neutrophils, *Cxcr6*, *Cxcr1*, and *Cxcr2* were identified as core receptors mediating recruitment, as they were predominantly expressed by NP2, the early‐infiltrating neutrophil subpopulation (Figure 4E). Within T cells, substantial heterogeneity was observed: *Ccr7* and *Cxcr4* were predominantly found in CD4+ T cells and Tregs (Figure 4F), whereas *Ccr5 and Cxcr3* were primarily expressed by CD8+ T cells, consistent with previous reports identifying them as key receptors mediating CD8+ T cell recruitment [35]. By comparing key receptor expression levels, we found that CD8+ T cells in the PLX group exhibited significant elevated *Ccr5* expression (Figure S5B), suggesting that this upregulation may compensate for the attenuated recruitment caused by reduced chemokine production, leading to an increased proportion of CD8+ T cells.

Subsequently, we focused on the infiltration of Tregs, which tend to accumulate in the injured brain over an extended period following stroke and contribute to the recovery of neurological function [36]. We then clustered Tregs into three subpopulations (Figure 4G). Treg 2 represented activated Tregs, characterized by high expression of *Itgae* and *Icos* (Figure 4H), consistent with previous reports [34]. Besides, Treg 2 also exhibited higher scores for pro‐repair and anti‐inflammatory functions (Figure S5C). Receptor expression analysis revealed cluster‐specific shifts; Treg 2 showed the most pronounced increase in *Cxcr4* expression in the PLX group, whereas Treg 1 showed elevated *Ccr4* levels compared to the CD group (Figure 4I). Correspondingly, microglia in the PLX group showed a notable upregulation of *Cxcl12* (the ligand for *Cxcr4*), while the expression of *Ccl17* and *Ccl22* (the ligand for *Ccr4*) remained unchanged (Figure 4B). These findings suggest that chemokine ligands may play a more pivotal role in guiding peripheral immune cell recruitment, and replenished microglia may facilitate the recruitment of activated Tregs.

By comparing the expression level of receptors between the two groups in MF, we found that despite increased *Ccr2* expression on infiltrating monocytes in the PLX group (Figure 4J), which is crucial for monocyte infiltration [37], the expression level of the corresponding chemokine ligand, Ccl2, exhibited a slight decrease on microglia (Figure 4B), suggesting that late‐phase increase in phagocytosis‐associated macrophages may result from local differentiation and proliferation.

Receptors *Cxcr2* and *Cxcr1*, which play key roles in neutrophil infiltration, were upregulated on NP2 in the PLX group (Figure 4K). However, the expression level of *Cxcl1* (the ligand for *Cxcr1*) on microglia showed no significant difference between the two groups (Figure 4B). However, *Cxcl2*, another key chemokine involved in the infiltration of neutrophils, was primarily expressed by neutrophils at 14 days post tMCAO (Figure S5D), consistent with previous findings [38], and showed a significant reduction in the PLX group (Figure S5E). These changes may underlie the decreased proportion of neutrophils in the PLX group.

Cytokine array analyses further confirmed a protein‐level reduction of several chemokines (CCL2, CCL3, CCL4, CCL21, CCL22) involved in recruiting CD4+ T cells in the PLX group, consistent with single‐cell findings (Figure 4L). Intriguingly, the elevated expression of CCL5 (Figure 4L), a key chemokine for CD8+ T cell recruitment (Figure 4F), might partially explain the increased proportion of CD8+ T cells observed in the PLX group.

Collectively, our results demonstrate that PLX5622 treatment modulates chemokine secretion by downregulating key ligands critical for peripheral immune cell recruitment. Notably, our findings suggest that chemokine ligands may play a more decisive role than receptors in regulating immune cell infiltration. This modulation effectively suppresses excessive immune cell infiltration, thereby promoting recovery during the chronic phase.

### 2.6 PLX5622 Treatment Promoted the Integrity of BBB by Inhibiting the Interaction Between Endothelium and Immune Cells

**FIGURE 5:**
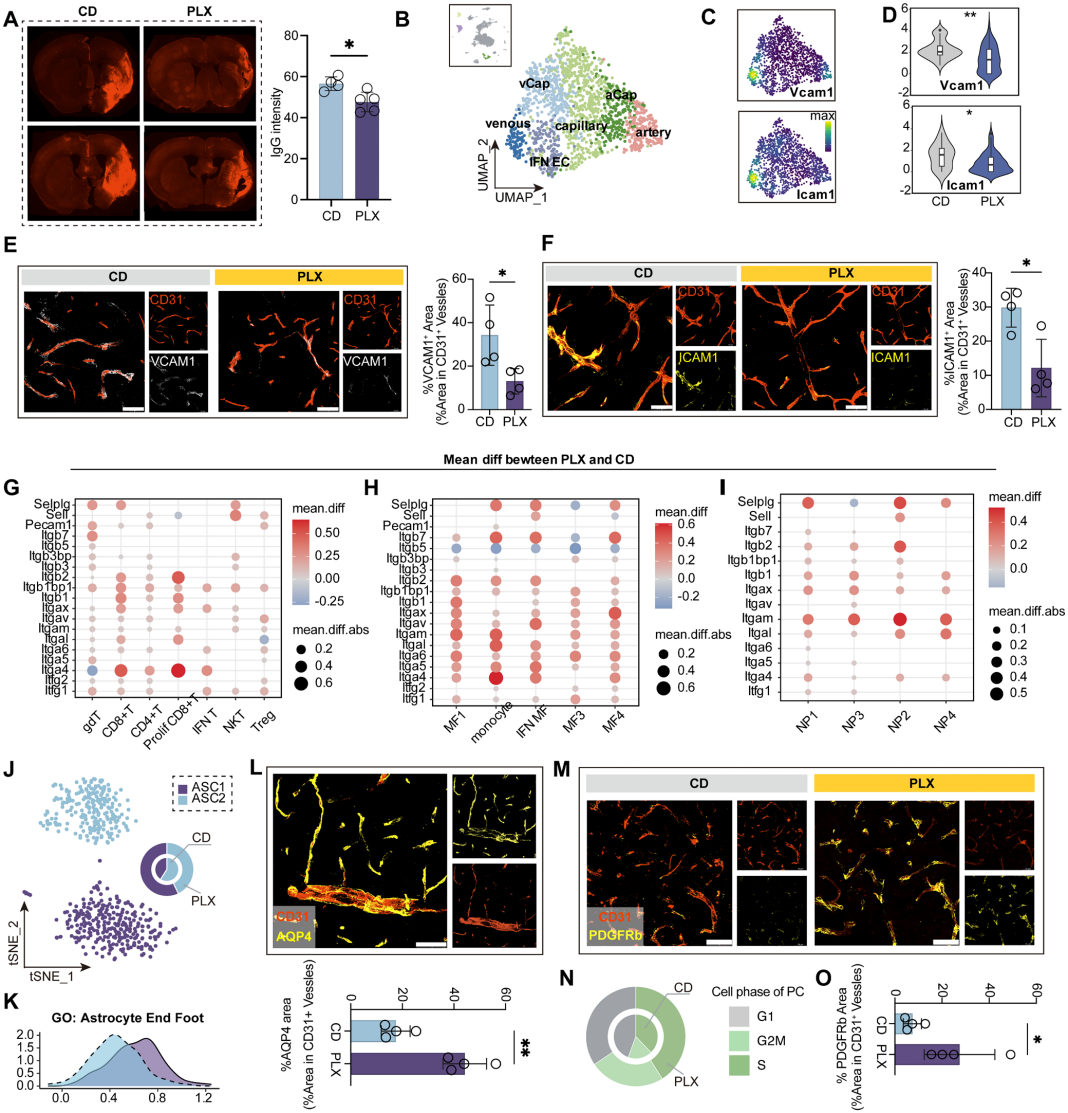
PLX5622 treatment promoted the integrity of BBB by inhibiting the interaction between endothelium and immune cells. (A) Representative plot showing Evan Blue extravasation (left). Quantification of tissue exudation with IgG (red) at 14 dpi in coronal sections (right). *n* = 4 for CD group, *n* = 5 for PLX group. Student's *t* test, two‐side, *p* = 0.0168. (B) UMAP projection of Endothelial cells. (C) Density plots in the UMAP space showing the expression Vcam1 and Icam1. Scale bar represents densities based on Kerenel density estimation of gene expression. (D) Violin plot showing the different expression level of Icam1 and Vcam1 between CD group and PLX group. *p < 0.05, **p < 0.01, Bonferroni adjusted *p*‐value by Wilcoxon rank sum test. (E) Representative confocal microscopic images of VCAM1 (white) and CD31 (red) (left), Scale bars, 50 μm., dot plots showing the quantification of VCAM1 colocalization with CD31 in each group (right). *N* = 4 for each group, Student's *t* test, two‐tailed. *p* = 0.0305. (F) Representative confocal microscopic images of ICAM1 (yellow) and CD31 (red) (left). Scale bars, 50 μm. dot plots showing the quantification of ICAM colocalization with CD31 in each group (right). *N* = 4 for each group, Student's *t* test, two‐tailed. *p* = 0.0131. (G–I) Changes in the mean expression levels of key adhesion associated molecular across T cells (G), MF (H) and neutrophil (I) subpopulations between the CD group and the PLX group. (J) tSNE projection of astrocytes. (K) Ridge plot showing the Module Score of gene set ‘Astrocyte End Foot’ across astrocyte subpopulations. (L) Representative images of AQP4 (yellow) colocalization with CD31 (red) (top), Scale bars, 50 μm. Dot plots showing the quantification of AQP4 colocalization with CD31 in each group (bottom). *N* = 4 for each group, Student's *t* test, two‐tailed. *p* = 0.0018. (M) Representative images of PDGFR‐β (yellow) colocalization with CD31 (red). Scale bars, 50 μm. (N) Donut charts illustrate the distribution of cell cycle phases (G1, G2/M, and S) within pericytes. (O) Dot plots showing the quantification of PDGFR‐β colocalization with CD31 in each group. *n* = 4 for each group, Student's *t* test, two‐tailed. *p* = 0.0412.

Apart from chemotaxis, the integral BBB represents another critical barrier limiting peripheral immune cell infiltration. To directly assess BBB integrity, Evans Blue was injected intraperitoneally to mice 30 min prior to sacrifice at 14 days post‐tMCAO. Early‐phase PLX5622 treatment reduced the area of Evans Blue exudation (Figure 5A), indicating a more intact BBB. Furthermore, IgG immunostaining revealed higher fluorescence intensity in the CD group, indicating reduced blood protein extravasation and improved BBB integrity in the PLX group (Figure 5A).

During the process of immune cell migration across the BBB, the interaction between endothelial cells and immune cells plays a crucial role [39]. To investigate this, we reanalyzed endothelial cells from our single cell dataset at 14 days post‐tMCAO and identified five subpopulations, including arterial, arteriole capillary (aCap), capillary, venule capillary (vCap), interferon‐associated endothelial (IFN EC), and venous (Figure 5B). Consistent with previous studies, Icam1 and Vcam1—key mediators of immune cell adhesion—were predominantly expressed in venous endothelial cells (Figure 5C) and were significantly downregulated in the PLX group (Figure 5D). Immunostaining confirmed these findings, showing decreased Vcam1 and Icam1 coverage in the PLX group (Figure 5E,F). Collectively, these changes suggest that early phase PLX5622 treatment contributes to diminished BBB‐immune cell interactions and suppresses the adhesion of peripheral immune cells.

Integrins and other adhesion molecules are essential mediators for immune cell‐endothelial interaction; thus, we compared their expression level between the two groups among major immune cells. Interestingly, we found an increased expression of adhesion molecules in peripheral infiltrating immune cells from the PLX group (Figure 5G–I). Within the T cells population, CD8+ T cells notably exhibited increased expression of adhesion molecules, including *Itga4* and *Selplg*, suggesting that stronger interaction between CD8+ T cells and endothelial cells may contribute to the increased proportion of them among T cells following PLX5622 treatment (Figure 5G).

For infiltrating MF and neutrophils, they displayed a general increase in integrin expression (Figure 5H,I). We speculate that during the migration process, endothelial cells may play a dominant role in mediating the infiltration of immune cells. Accordingly, in the PLX group, the reduced expression of Icam1 and Vcam1 on endothelial cells (Figure 5C–F) may necessitate higher adhesion molecule expression in peripheral immune cells to facilitate infiltration.

Moreover, we observed a significant increase in neurovascular unit components—including pericytes, astrocytes, and endothelial cells—in the PLX group (Figure S1D). Notably, the role of astrocytes (forming the glia limitans) and pericytes in maintaining BBB integrity has gained increasing attention [40, 41]. We then conducted a deeper investigation into astrocytes and identified two distinct subpopulations, ASC1 and ASC2 (Figure 5J). The proportion of ASC1 was significantly increased in the PLX group (Figure 5J). Between the two clusters, ASC1 exhibited higher scores for astrocytic end foot formation (Figure 5K), a process critical for the structure and function of glia limitans [42], suggesting that PLX5622 treatment may enhance glia limitans formation. Immunostaining for AQP4 (the key molecule involved in astrocyte end foot formation) and CD31 (Figure 5L) was then performed. The result confirmed increased astrocytic end foot coverage on endothelial cells following PLX5622 treatment (Figure 5L), suggesting that early‐phase PLX5622 administration enhances glia limitans formation and contributes to the protection of BBB integrity.

For pericytes, we observed a significant increase in the proportion of proliferating clusters (Figure 5N), which may contribute to enhanced endothelial coverage and support BBB integrity. To validate this, we performed immunostaining for CD31 and PDGFRβ (Figure 5M), which further confirmed increased pericyte coverage in the PLX group (Figure 5O). Furthermore, pericyte clustering identified a subpopulation expressing *Acta2*, indicative of a transformation toward smooth muscle‐like cells [43], which showed a slight increase in the PLX group (Figure S6B). These cells are involved in regulating microvascular constriction and may influence stroke prognosis by modulating blood flow [43]. However, their specific role warrants further investigation. Collectively, increased coverage of pericytes and astrocyte end foot suggests enhanced BBB integrity after early‐phase therapy of PLX5622.

Various factors contribute to maintaining BBB integrity; among these factors, the role of immune cells has attracted increasing attention [38, 44, 45]. We then performed CellChat analysis to explore immune‐endothelial interaction, revealing that microglia exhibited stronger interaction weights with astrocytes and pericytes in the PLX group compared to other immune cell types (Figure S6B). We also evaluated BBB integrity 3 days post tMCAO, which is the peak time of inflammatory BBB disruption [46]. We found that microglia depletion during the acute phase did not affect BBB permeability, as shown by comparable IgG leakage between mice on PLX5622 diet and those on control diet (Figure S6C). In contrast, a significant decrease in IgG extravasation was observed in the PLX group vs. CD group at 14 days after stroke (Figure 5A). This finding suggests a potential direct interaction between repopulated microglia and the BBB. Therefore, we further focused on investigating microglia‐BBB interactions. By applying CellChat interaction analysis, we found that in the PLX group, the binding capacity between microglia and astrocytes was enhanced, as indicated by increased *Cadm1*‐mediated interactions. Additionally, the expression of *Psap*, which activates the neuroprotective and glioprotective receptor *Gpr37l1* on astrocytes, was elevated (Figure S6D). These interactions enhance astrocytes' ability to resist oxidative stress and cell death [47]. For pericytes, the *Spp1* signaling axis (which encodes osteopenia, a molecule that limits inflammation and promotes axon regeneration) and the *Tgfb1* signaling axis were markedly enhanced in the PLX group, indicating that replenished microglia can promote the proliferation of pericytes, thereby increasing their coverage and contributing to BBB stabilization (Figure S6D).

In summary, the immune microenvironment reshaped by PLX5622 treatment influenced BBB composition and function. The downregulation of endothelial adhesion molecules (*Vcam1* and *Icam1*), along with increased proliferation and coverage of astrocytes and pericytes, contributed to improved BBB integrity.

### 2.7 Proliferative Capacity and Functional Reprogramming of Peripheral Immune Cells Following PLX5622 Treatment

**FIGURE 6:**
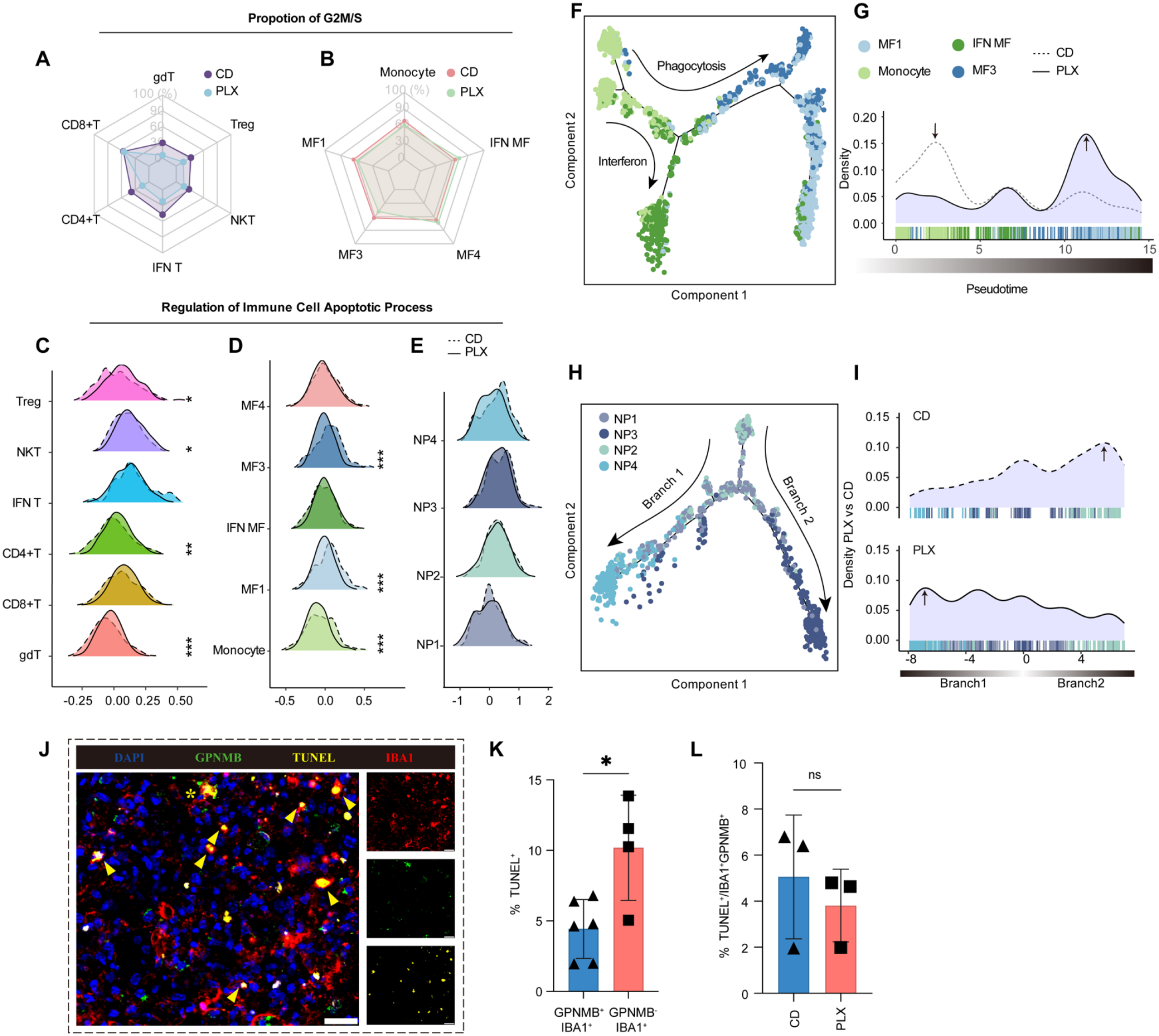
The ability of proliferation and function transition of peripheral immune cells after PLX5622 treatment. (A, B) Radar plots illustrating the proportion of cells in the G2/M and S phases across T cells and MF subpopulations in the CD and PLX groups. (C–E) Ridgeline plot summarizing the apoptosis process of main immune cell (T cells (C), MF (D), neutrophil (E)) at day 14, measured by module scores. *p < 0.05, **p < 0.01, ***p < 0.005. Bonferroni adjusted *p*‐value by Wilcoxon rank sum test (F) Inferred differentiation trajectory of MF in Brach 2 with monocle2. Arrows indicate the developmental directions of interferon and phagocytosis properties. (G) the distribution of MF in branch 2 on pseudotemporal axes in the indicated groups. Gray dash line, the density curves of CD group. Color‐coded bars indicated the positions of MF on pseudotemporal axes. (H) Inferred differentiation trajectory of neutrophil with monocle2. Arrows indicate the developmental directions of interferon (Branch 1) and apoptosis (Branch 2) properties. (I) the distribution of neutrophil on pseudotemporal axes in the indicated groups. Color‐coded bars indicated the positions of neutrophil on pseudotemporal axes. (J) Representative images of IBA1 (red), GPNMB (green), TUNEL (yellow), DAPI (blue). Arrows indicate double‐positive cells for IBA1 and TUNEL. Asterisks indicate double‐positive cells for IBA1 and GPNMB. Scale bars, 20 μm. (K) Dot plots showing the quantification of TUNEL^+^ proportion in IBA1^+^GPNMB^+^macrophages and IBA1^+^GPNMB^−^macrophages. Scale bars, 20 μm *n* = 6 for GPNMB^+^IBA1^+^ macrophage, *n* = 4 for GPNMB^−^IBA1^+^ macrophage, Student's *t* test, two‐tailed. *p* = 0.0134. (L) Dot plots showing the quantification of TUNEL^+^ proportion in IBA1^+^GPNMB^+^macrophages between the two groups. *n* = 3 for each group, Student's *t* test, two‐tailed. *p* = 0.5267.

The composition of infiltrating immune cells after ischemic stroke is shaped not only by their ability to infiltrate but also by the brain's immune microenvironment, which governs their proliferation/apoptosis and differentiation. Cell cycle scoring was used to evaluate the proliferative potential of T cells and MF (Figure 6A,B). We found that, except for CD8+ T cells, which showed no significant change in the proportion of cells in the G2/M and S phase, other T cell subpopulations, particularly CD4+ T cells and gamma‐delta T (gdT) cells, exhibited a reduced proliferation ratio in the PLX group (Figure 6A), while for MF, no significant proliferation changes were observed (Figure 6B). Thus, we speculate that decreased proliferative capacity may contribute to the decreased proportion of CD4+ T cells in the PLX group.

Additionally, apoptosis also plays a key role in regulating immune cell populations. Apoptosis‐related gene signature scoring revealed no significant differences among neutrophil subpopulations (Figure 6E). However, CD4+ T cells, Tregs, NKT cells, and gamma‐delta T (γδT) cells showed increased apoptosis levels in the PLX group (Figure 6C), whereas monocytes, MF1, and MF3 displayed reduced apoptosis scores (Figure 6D). The observed differences in apoptosis levels may have played a role in shaping the eventual composition of immune cell subpopulations. Furthermore, immunofluorescence staining revealed that GPNMB^+^IBA1^+^ cells exhibited a lower apoptotic rate compared to GPNMB^−^IBA1^+^ cells, suggesting that differentiation into GPNMB^+^ macrophages may confer enhanced resistance to apoptosis (Figure 6J, K ). However, the apoptotic ratio of GPNMB^+^ macrophages did not differ significantly between the two groups (Figure 6L ). We propose that the discrepancy between immunostaining and scRNA sequencing may stem from temporal differences between RNA and protein expression; therefore, additional time‐course analyses may be necessary in future studies.

Since monocytes and neutrophils can differentiate into distinct subpopulations in response to environmental cues, variations in differentiation trajectories will also influence the fate and transformation of these infiltrating immune cells. Using Monocle3, we identified two major differentiation trajectories for infiltrating monocytes: Branch 1 gives rise to antigen‐presenting macrophages, while Branch 2 leads to interferon‐associated or phagocytosis‐related macrophages (Figure S7A). Following early‐phase therapy of PLX5622, monocytes preferentially differentiated into interferon‐associated and phagocytosis‐related macrophages. Then, to identify key factors promoting differentiation toward Branch 2 in the PLX group, we analyzed DEGs across subpopulations and found 38 genes specifically upregulated in Branch 2 (Figure S7B). Among them, *S100a4*, a gene critical for chemotaxis [48], migration [49], and differentiation into M2‐like macrophages [50], exhibited markedly elevated expression in the PLX group (Figure S7C). Further analysis of Branch 2 with Monocle2 confirmed increased differentiation into phagocytosis‐related macrophages in the PLX group (Figure 6F,G).

Neutrophil differentiation trajectories were then analyzed using Monocle2, revealing two distinct paths: Branch 1 leading to interferon (IFN)‐associated neutrophils and Branch 2 toward terminally differentiated neutrophils (Figure 6H). Notably, neutrophils in the PLX group predominantly followed Branch 1 (Figure 6I), indicating that the reshaped microenvironment can suppress terminal differentiation of infiltrating neutrophils. Regulatory network analysis using pySCENIC identified *Klf6*—a key regulator of neutrophil maturation and terminal differentiation [51]—and *Maff*, which is associated with inflammation [52], as the core transcription factors driving the differentiation of neutrophils to NP3 (Figure S7D). Conversely, *Irf7* and *Stat1*, key transcription factors involved in interferon signaling, were enriched in NP4, consistent with their interferon‐associated functional profile (Figure S4I). Thus, the reshaped microenvironment induced by PLX5622 treatment appears to modulate neutrophil transcriptional activity by suppressing factors associated with terminal differentiation (*Klf6*, *Maff*) while enhancing interferon‐associated transcriptional programs (*Irf7*, *Stat1*) (Figure S7E).

Overall, the microglia repopulation after ischemic stroke not only modulates peripheral immune cell infiltration but also directs their differentiation away from pro‐inflammatory phenotypes and toward protective, anti‐inflammatory states, thereby contributing to improved neurological outcomes.

## 3 Discussion

The prognosis of ischemic stroke is influenced not only by the initial ischemic insult but also by secondary damage resulting from sustained neuroinflammation [53, 54]. In our study, we propose a novel therapeutic approach—transient microglia depletion during the acute phase using PLX5622, followed by repopulation during the subacute phase. This strategy restored microglial homeostasis, created a more stable microenvironment, strengthened BBB integrity, and effectively inhibited the infiltration of peripheral immune cells. Additionally, the reshaped immune microenvironment facilitates beneficial phenotypic transitions in infiltrating immune cells, thereby promoting neurological recovery. This time‐targeted modulation of microglia offers a promising and clinically translatable strategy for stroke treatment.

In our study, we found that repopulated microglia exhibited phenotypic similarities to microglia in the CD group, supporting recent findings that repopulated microglia originate exclusively from residual microglia [55]; we inferred that the replenished microglia arose from self‐proliferation of residual microglia, as they exhibited similar expression levels of Sall1 and Sall3‐ markers specific to yolk sac‐derived microglia [25, 26]‐compared to control microglia. While definitive confirmation of these repopulated microglia's origin will require fate‐mapping models such as Tmem119‐Cre mice in future studies.

Recent findings show that peripheral macrophages can infiltrate the brain and acquire microglia‐like phenotype [30, 56]. While compared to bona fide microglia, these microglia‐like cells exhibited higher expression of *Apoe*, *Lyz2*, and *Ms4a7*, as they cannot completely acquire the microglia phenotype [25, 26]. In our study, we also found a cluster of microglia‐like cells that highly expressed *Ms4a7*, *Apoe*, and *Lyz2*, and had a higher score of peripheral infiltration macrophage, suggesting peripheral derivation. However, the origin of these Ms4a7+ MG, their long‐term fate—whether they undergo apoptosis or persist—and their role in shaping the chronic brain microenvironment remain to be further elucidated. Fate‐mapping models such as Ms4a3‐Cre [57] or Flt3‐Cre [30] mice, which enable tracing of bone marrow‐derived macrophages, should be employed in future studies to clarify their origin and dynamics.

Previous studies have demonstrated that microglia repopulation after brain injury improves functional recovery [58, 59]. However, they lack in‐depth investigation into subtype transitions. In our study, early‐phase microglia attenuation after stroke resulted in an increased proportion of homeostatic microglia and a reduction in pro‐inflammatory subtypes, consistent with recent findings that microglia repopulation helps normalize proinflammatory signaling [60]. The mechanism by which the repopulated microglia maintain a more homeostatic‐like state remains unclear. It is known that multiple signals, such as DAMPs, are released from the injured brain after stroke, leading to the activation of immune cells and subsequent release of inflammatory factors. All these noxious stimulations propagate microglial activation and disturb their homeostasis [61]. In our study, early PLX5622 treatment may deplete the overactivated microglia and attenuate poststroke inflammation (Figure 1). This milder inflammatory milieu may prevent excessive microglia activation and maintain the repopulated microglia in a more homeostatic state. Moreover, accumulating evidence suggests that infiltrating immune cells also play a regulatory role in microglia phenotype transitions [62]. In our study, the reduced infiltration of peripheral immune cells and an increased presence of reparative subpopulations following PLX5622 treatment may further facilitate this transition. B cell depletion has been shown to inhibit microglia activation [32]. Similarly, we observed a significant reduction in B cells after microglia repopulation, which may contribute to preventing the transition of microglia toward a pro‐inflammatory phenotype. In summary, the shift of repopulated microglia toward a homeostatic phenotype involves complex and coordinated extrinsic environmental cues and intrinsic regulatory mechanisms, warranting further mechanistic studies.

Infiltrating immune cells undergo dynamic functional shifts after stroke, and restoring immune homeostasis is crucial for recovery [7]. In our study, acute‐phase PLX5622 treatment led to a reduction in neutrophils, B cells, and T cells during the later phase, and profoundly reprogrammed their phenotypes and functions. This resulted in a decrease in pro‐inflammatory phenotypes and an increase in repair‐associated subsets. However, among the infiltrating T cells, we observed an increased proportion of CD8+ T cells in the PLX group. Numerous studies have shown that chemotactic recruitment [63] and clonal expansion [64] exert critical roles in regulating CD8+ T cell infiltration. In our study, we observed significantly elevated Ccr5 expression and an increased proportion of proliferative CD8+ T cells in the PLX group. These findings suggest that both enhanced chemotactic signaling and clonal proliferation may contribute to the increased CD8+ T cell ratio in T cells observed after PLX5622 treatment. Nonetheless, additional investigations—such as TCR sequencing and in vivo functional validation—are warranted to substantiate these proposed mechanisms. Previous studies have shown that reducing CD8+ T cell recruitment improves functional recovery in the late stages of stroke [65], suggesting a negative association with prognosis. However, recent evidence indicates that brain resident CD8+ T cells exert protective effects by suppressing microglia activation via the CXCL16‐CXCR6 axis in Alzheimer's disease [66]. In our study, we observed an increase in Cxcr6 expression of CD8+ T cells in the PLX group; this may indicate an anti‐inflammatory role, while the precise role of CD8+ T cells in the reshaped microenvironment requires further investigation.

The integrity of BBB undergoes dynamic changes after stroke. During the acute phase (within 3 days), the BBB primarily experiences disruption in response to neuroinflammation [46]. Subsequently, starting from the subacute phase (1–3 weeks), repair mechanisms take place [67]. Among this process, both the inflammatory microenvironment and interactions with glial and immune cells [62, 68] reshape BBB integrity. Recent studies have shown that A2‐type astrocytes [69] and pericyte [70] participate in maintaining BBB integrity. In addition, interactions with immune cells, such as macrophages [45, 71], neutrophil [72], CD4^+^ T cells [73], and B cells [74], also contribute to the process. In our study, we focus on the interaction between microglia and BBB, as microglia exhibited the greatest increase in interaction weight and have been shown to play a key role in regulating BBB integrity [45, 75]. While further studies are needed to elucidate how the remodeling of other immune cells affects BBB integrity. Nevertheless, some studies have challenged the protective role of astrocytic end feet [76]. Meanwhile, the increased α‐SMA^+^ pericytes indicate enhanced contractility, which might be associated with reduced cerebral blood flow following stroke [77, 78]. Therefore, further studies using genetic tools are needed to clarify their contributions to BBB maintenance and stroke recovery.

In conclusion, our novel therapeutic approach effectively reshaped the immune microenvironment within the mouse brain following ischemic stroke at the chronic stage. We observed a significant reduction in immune cell infiltration and an attenuated inflammatory environment. Furthermore, our findings revealed that transitioning microglia toward a homeostatic phenotype, reducing chemokine production, and enhancing blood–brain barrier (BBB) integrity are crucial mechanisms in limiting immune cell infiltration and maintaining immune stability. Collectively, our study provides novel insights into a potential treatment strategy for ischemic stroke by modulating the brain's immune microenvironment.

## 4 Limitation

PLX5622 not only depletes brain resident microglia but also induces long‐term system alterations [24]. It affects both tissue resident and circulating myeloid and lymphoid compartments, as reflected in our observation of reduced peripheral monocytes post‐treatment. Additionally, PLX5622 has been shown to alter cholesterol metabolism in brain endothelial cells independently of microglia depletion [79] and cause systemic metabolic disturbances [80], which may confound experimental outcomes. Therefore, cell‐specific genetic models should be further employed to better elucidate the effects of microglia repopulation on stroke. The clinical relevance of our findings in mice warrants further validation in human studies. Additionally, most of the findings were primarily based on single‐cell level analyses and lacked corresponding experimental validation. Future studies are needed to investigate these alternative mechanisms. In addition, recent studies have shown that sex influences prognosis following microglia repopulation in a TBI model [59], suggesting that sex differences should also be taken into account in future analyses.

## 5 Methods

### 5.1 Animals

Young (8–12 weeks old) male C57BL/6 mice obtained from SLAC Laboratory Company (Shanghai, China) were used for in vivo experiments. All animals were housed in environmentally controlled cages with appropriate temperature and humidity, adhering to a 12‐h light/dark cycle, and provided ample food and water. Animals were randomly assigned to either the sham or stroke groups and received treatments determined by a lottery drawing method. All treatments and analyses were conducted by blinded investigators.

### 5.2 Transient Cerebral Ischemia Model

Transient cerebral ischemia models were established by endovascular occlusion of the left middle cerebral artery (MCA) for 60 min. Briefly, mice were anesthetized with 1% Pentobarbital and maintained on a 30% O_2_/70% N_2_O mixture to ensure spontaneous breathing. A filament with a silicon‐coated tip (RWD, Shenzhen, China) was inserted into the external carotid artery (ECA). After adjusting the direction into the internal carotid artery (ICA), the filament was advanced to the origin of the MCA until resistance was felt. The filament was then left in position for 60 min to obstruct cerebral blood flow. After this period, the filament was withdrawn to restore brain blood flow, and the residual end of the ECA was ligated. The body temperature of the mice was maintained at 37.0°C ± 0.5°C during the operation, and the mice were placed on a water bath blanket until they woke up from anesthesia. The sham group animals received the same anesthesia and experienced carotid triangle exposure without left MCA occlusion. The surgeons were blinded to the grouping of the experimental animals. For analgesia, mice were injected intraperitoneally with Ketoprofen (3 mg/kg body weight) before surgery and once a day for three consecutive days after surgery. The mortality rates in this study are listed in Table S2.

### 5.3 Behavior Tests

We conducted a series of behavioral tests to assess neurofunctional deficits in rodents following stroke. The behavioral tests and statistical analyses were conducted by different researchers, ensuring blinding to treatment throughout the experiments. All data were expressed as mean values from three repeated trials per day.

### 5.4 Adhesive Removal Test

In this test, a 2 × 3 mm adhesive tape was applied to the right forelimb of the mice. We recorded the time taken to perceive and remove the tape separately to assess tactile response and sensorimotor asymmetry. The maximum observation window for perception was 60 s; for removal, it was 120 s.

### 5.5 Foot Fault Test

Briefly, mice tightly grasped the wire while moving on a wire fence with a grid size of 2 × 2 cm located 1 m above the ground. A “foot fault” was defined as a slip of the paw through the grid due to motor impairment. We recorded the percentage of forelimb contralateral to the injured hemisphere's falling times compared to the total of fifty steps to evaluate locomotor function.

### 5.6 CSF1R Inhibition

For early‐phase CSF1R inhibition, mice were provided with PLX5622 (Chemgood) in the diet (Research Diets) at a concentration of 1200 PPM (1200 mg/kg of chow) and were intragastric administration of PLX5622 at a dose of 90 mg kg^−1^d^−1^ [81]. The administration of PLX5622 chow began on the day of MCAO and continued until 7 dpi.

### 5.7 Cytokine Array

Cytokine array was conducted according to the documented protocol [82]. Briefly, brain homogenates were prepared from MCAO mice 14 days after surgery, which had been treated with or without PLX5622 for 7 days. After the total protein concentration was adjusted to 1 mg/mL, cytokine levels in these samples were detected using a Mouse XL Cytokines Array Kit (R&D Systems Inc. Minneapolis, MN) according to the manufacturer's instructions.

### 5.8 Immunostaining of Brain Sections

Coronal brain slices were utilized for immunostaining. In brief, the floating brain slices were washed twice with PBS, followed by treatment with 0.5% Triton‐X in PBS (PBST) for 15 min to permeabilize cell membranes at room temperature. Subsequently, the slices were rinsed three times with 0.3% PBST for 5 min each. They were then blocked with 5% normal donkey serum in 0.3% PBST at room temperature for 1 h before overnight incubation at 4°C with the following primary antibodies: Goat anti‐CD31 (R&D, AF3628, 1:200), Goat anti‐IBA1 (Abcam, ab5076, 1:250), Mouse anti‐AQP4 (Santa Cruz, sc‐390488,1:100), Rat anti‐VCAM1 (Invitrogen,14‐1061‐82, 1:200), Rat anti‐ICAM1 (Invitrogen, 14‐0542‐82, 1:200), Goat PDGFRB (R&D, AF1042, 1:50), Rat anti‐MBP (Abcam, ab7349, 1:250), Rabbit anti‐GPNMB (Abcam, ab188222, 1:200), Rabbit anti‐MAP2 (Proteintech, 17490–1‐AP, 1:500), anti‐mouse IgG‐Alexa Fluor 488 (CST, 4408S, 1:200), Rabbit anti‐TMEM119 (Abcam, ab209064, 1:200). For the apoptosis test, one‐step TUNEL apoptosis assay kit (Beyotime, C1090) was used After washing with 0.3% PBST three times for 10 min each, brain slices were incubated with appropriate secondary antibodies conjugated with Alexa Fluor 488, 555, 594, and/or 647 (Invitrogen, 1:500) in a dark environment at room temperature for 1 h. Subsequently, brain slices were washed with PBS three times and mounted on glass slides with mount‐G containing DAPI (Yeasan Biotech). The Leica TCS SP8 confocal microscope (Leica Microsystems) was employed to observe and capture images of the sections.

### 5.9 Measurement of Tissue Loss

Brain samples were sectioned with a cryo‐microtome into 6 evenly spaced layers of coronal slices (25 μm thick) and then stored in a cryoprotectant solution (40% PBS, 30% ethylene glycol, 30% glycerol) at −20°C until immunostaining. For each layer, one brain section slice was chosen and stained with an anti‐MAP2 antibody. Tissue loss was assessed in six equally spaced 25 μm coronal brain sections stained for MAP2, spanning approximately 1.10 mm anterior to bregma to 2.06 mm posterior to bregma.

### 5.10 Flow Cytometry

To obtain a single‐cell suspension of intracerebral immune cells, mice were euthanized, and transcardial perfusion was performed using 25 mL of pre‐cooled PBS. Left hemispheres were collected after brain dissection. Brain tissues were dissociated into homogenates using the Neural Tissue Dissociation Kit (T) with a gentleMACS Octo Dissociator with Heaters (Miltenyi Biotec), following the manufacturer's instructions. The resulting suspension was passed through a 70 μm cell strainer, and the resulting cells were resuspended in 10 mL of 30% Percoll. Subsequently, 3 mL of 70% Percoll was slowly injected into the bottom of the suspension using a fine needle, while 1 mL of PBS was layered on top of the suspension. Centrifugation (800 g, 30 min, 18°C) was performed using a density gradient of 30%/70% Percoll to separate myelin sheaths and debris. Cells at the interface, mainly consisting of immune cells, were collected, washed, and resuspended in pre‐cooled PBS. For blood cell preparation, mice were anesthetized, and peripheral blood was collected from the right atrium. The final 2 mL blood suspension (0.8 mL blood +1.2 mL PBS) was layered onto 1 mL of Ficoll‐Paque (GE, 17–1440‐02). Cells at the interface were collected after density gradient centrifugation (800 g, 20 min, 18°C) and then resuspended with pre‐cooled PBS. The single‐cell suspension obtained as described above was incubated with antibodies to surface antigens in the dark for 30 min on ice at 4°C. Fluorochrome compensation was established with single‐stained UltraComp eBeads (Thermo Fisher Invitrogen). Flow cytometry was performed using the BD LSRFortessa flow cytometer (BD biosciences). FlowJo software was used for data analysis. The antibodies used for flow cytometry included: anti‐CD45‐Pacific blue (Biolegend, 103126), anti‐CD11b‐PE‐CY7 (Biolegend, 101216), anti‐Ly6G‐PerCP‐Cy5.5 (Biolegend, 127616), anti‐CD19‐PE (Biolegend, 115508), anti‐NK1.1‐BV605 (Biolegend, 108740), anti‐CD3e‐FITC (BD, 553061), anti‐CD4‐APC‐CY7 (BD, 552051), anti‐CD8a‐APC (BD, 553035), anti‐GPNMB‐eFluor660 (Thermo Fisher Scientific, 50–5708‐82), anti‐CD115‐BV605 (Biolegend, 13355174) (see Table S1 for details).

### 5.11 Single Cell RNA Sequencing

Single cell suspensions were prepared according to our previously published work [36, 44]. In brief, animals were euthanized and subjected to ice‐cold saline perfusion at either 5‐ or 14‐days post MCAO. Ischemic hemispheres were collected, excluding meninges, the olfactory bulb, or cerebellum. For the scRNA‐seq analysis of entire brain cells, brain homogenates were created using the Adult Brain Dissociation Kit (T) with a gentle MACS dissociator with heaters (Miltenyi Biotec), following the provided guidelines. In the case of scRNA‐seq for CD45high immune cells, brain homogenates were generated using the Neural Tissue Dissociation Kit (T), and CD45^high^ immune cells were isolated through FACS using the anti‐CD45‐PE‐Cy5 antibody (Thermo Fisher Scientific, 15–0451‐81). For PBMC specimens, peripheral blood was collected from the right atrium, followed by two rounds of red blood cell lysis (eBioscience, 1X RBC lysis buffer, 00–4,333,057). Libraries for 10X Genomics were prepared using the 10X Genomics Chromium Single Cell 3′ v3 chemistry, in accordance with the manufacturer's instructions. Subsequently, the mixture containing captured and barcoded mRNAs was retrieved from the Chromium instrument, followed by reverse transcription. The cDNA samples were fragmented and amplified per the 10X protocol. The libraries were then purified, quantified, and sequenced utilizing the Illumina NovaSeq sequencer.

### 5.12 Preprocessing and Clustering Analysis of Single‐Cell Transcriptome Data

The fundamental processing and visualization of the scRNA‐seq data were carried out using the Seurat package (version 5.0.1) in R (version 4.3.2) [83]. Briefly, low‐quality cells and doublets were filtered out based on the following criteria: (i) the number of expressed genes was less than 200 or more than 6000 and (ii) the percentage of mitochondrial genes was more than 10%. Then data normalization and variance stabilization were carried out with the SCTransform function. The batch effect was then removed using the harmony function [84]. After principal component analysis (PCA), the FindClusters function was applied to identify different clusters, followed by nonlinear dimensional reduction methods used for visualization, including uniform manifold approximation and projection (UMAP) and t‐distributed Stochastic Neighbor Embedding (tSNE). Clusters expressing marker genes from multiple cell types or with low gene content were identified as low‐quality or doublets and were excluded from further analysis. For each subpopulation, identical analyses were performed after individual extraction.

### 5.13 Differentially Expressed Gene Calculation

The Seurat FindAllMarkers and FindMarkers functions were employed to identify differentially expressed genes (DEGs) using the Wilcoxon rank sum test. Specifically, genes with an adjusted Bonferroni P‐value of less than 0.05 and an absolute log2 (fold change) value greater than 0.25 were considered DEGs.

### 5.14 Functional Enrichment Analysis

Gene Ontology (GO) enrichment analyses were performed on Metascape (http://metascape.org) [85]. A list of DEGs was uploaded, and statistically enriched GO terms were returned. *Z*‐score was calculated with the GOplot package in R to predict the activation state for each GO term. A term was considered to be significantly changed with a *Z*‐score > 2 or < −2 and an adjusted *p* value < 0.01.

### 5.15 Cell Phase Classification

The CellCycleScoring function in the Seurat R package was employed to classify cells into G2M, S, or G1 phases based on the expression of cell‐phase specific genes.

### 5.16 Pseudotime Analysis

Monocle (version 2.30.1), Monocle3 (version 1.3.4) R packages were employed for pseudotime analysis.

### 5.17 Calculation of Functional Gene Set Module Score

To quantify biological functions, the AddModuleScore method was applied to calculate the average expression levels of each specific gene set on a single cell level, subtracted by the aggregated expression of control gene sets. All analyzed genes are binned based on averaged expression, and the control genes are randomly selected from each bin. The gene sets were constructed based on the MSigDB molecular signature database (https://www.gsea‐msigdb.org/gsea/msigdb/index.jsp) and published work [34, 36, 86, 87]. Gene sets were listed in Table S3.

### 5.18 Cell–Cell Interaction Analysis

The CellChat (version 1.6.1) R package was applied to infer intercellular communication. Briefly, DEGs calculated as previously described were loaded, and the official workflows were run in the R tool. The probability of interactions within each cluster was calculated and further grouped by signaling types.

### 5.19 Single‐Cell Regulatory Network Inference

pySCENIC package (v.0.12.1) was conducted to identify different transcription factors (TFs) and discover regulons [88]. The mc9nr databases and their corresponding motif annotations were utilized to infer the gene regulatory network within individual cells. Subsequently, a heatmap of normalized regulon AUC was generated using R.

### 5.20 Statistical Analysis

Results are presented as mean ± standard deviation (SD). The scRNA‐seq data were statistically analyzed using the Wilcoxon rank sum test. Statistical comparison of the means between two groups with equal variances and normal distributions was performed by using the student's *t*‐test. Differences in means across groups with repeated measurements over time were analyzed using the two‐way repeated‐measures ANOVA or mixed‐effects model. All statistical analyses were performed with R (version 4.3.2) or GraphPad Prism (version 10.1.1). Statistical significance was defined as *p* < 0.05. All the data were quantified by two independent observers blinded to grouping.

## Ethics Statement

Ethical approval (Approval no. 2020‐655) for all animal experimental procedures was granted by the Institutional Ethics Committee of the Second Affiliated Hospital, Zhejiang University School of Medicine, and conformed to the guidelines outlined in the National Institutes of Health Guide for the Care and Use of Laboratory Animals.

## Conflicts of Interest

The authors declare no conflicts of interest.

## Supporting information


**Figures S1–S7:** cns70565‐sup‐0001‐FiguresS1‐S7.docx.

**Table S1:** cns70565‐sup‐0002‐TableS1.xlsx.

**Table S2:** cns70565‐sup‐0003‐TableS2.xlsx.

**Table S3:** cns70565‐sup‐0004‐TableS3.xlsx.

**Table S4:** cns70565‐sup‐0004‐TableS4.xlsx.

## Data Availability

The data that support the findings of this study are available from the corresponding author upon reasonable request.
