# Supplementary material for: Repopulating Microglia Suppress Peripheral Immune Cell Infiltration to Promote Poststroke Recovery
Source: CNS Neurosci Ther. 2025 Sep 10;31(9):e70565. doi: 10.1111/cns.70565 (PMC12423550; doi:10.1111/cns.70565)
Supplement: Supplementary file 1 — Figures S1–S7: cns70565‐sup‐0001‐FiguresS1‐S7.docx. [file CNS-31-e70565-s002.docx]

Supporting Information

**Repopulating Microglia Suppress Peripheral Immune Cell Infiltration to Promote Post-Stroke Recovery**

Ligen Shi^1,2,3^*, Lingxiao Lu^1,3^*, Jun Hu^1^*, Jiarui Chen^1,3^, Qia Zhang^1,3^, Ziyang Jin^1,3^, Zhen Wang^1^, Zhe Zheng^1,3#^, and Jianmin Zhang^1,2,3#^.

^1^ Department of Neurosurgery, Second Affiliated Hospital, School of Medicine, Zhejiang University, Hangzhou, Zhejiang, China;

^2^ Research Center for Life Science and Human Health, Binjiang Institute of Zhejiang University, Hangzhou 310053, China;

^3^ Clinical Research Center for Neurological Diseases of Zhejiang Province, Hangzhou, China.

* The authors contributed equally.

**This file includes:**

Supplementary FigureS1-S7

**Supplementary Figures:**


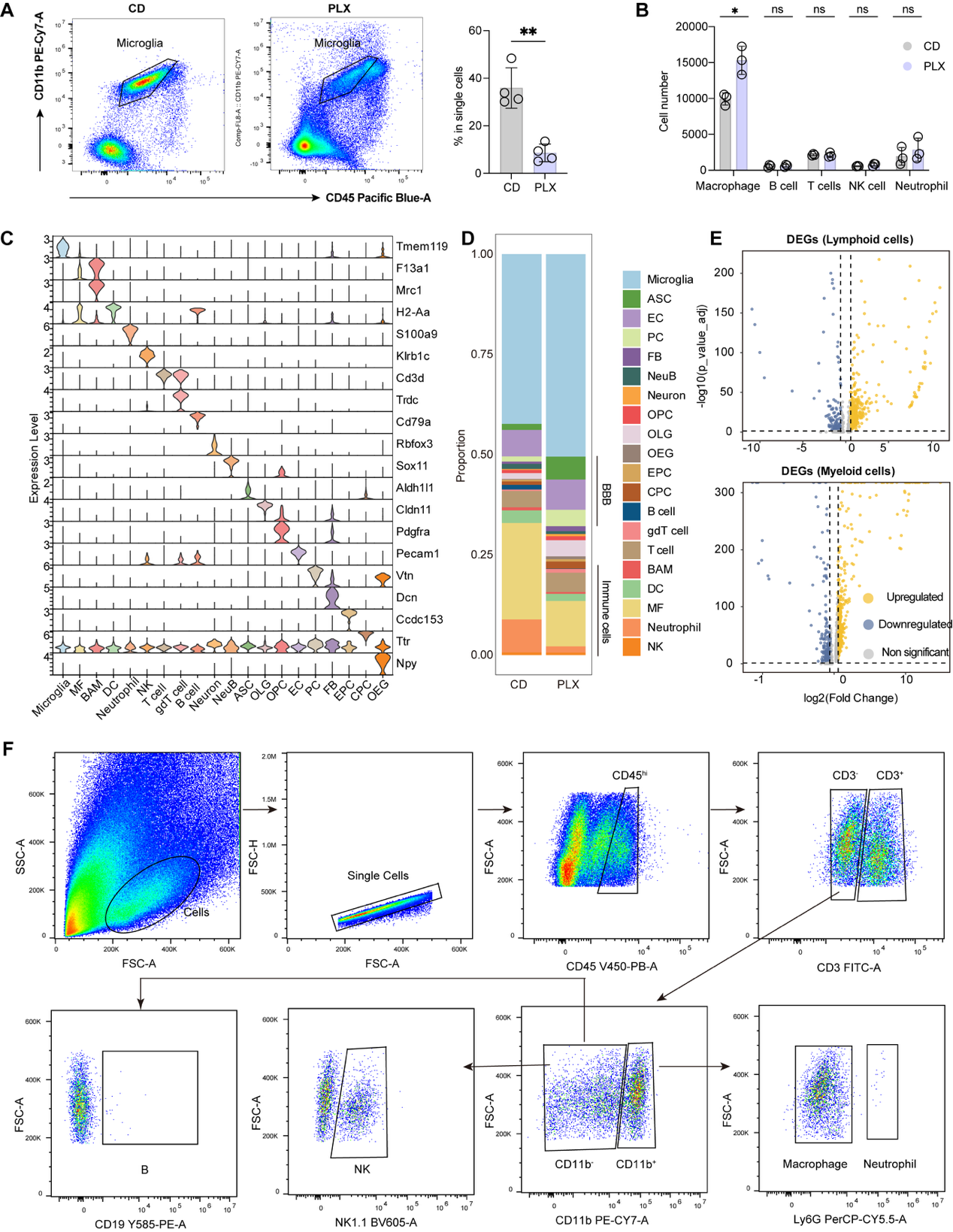


**Supplementary Figure 1:** A) Flow cytometry was performed to quantify the numbers of microglia at 7 days post tMCAO (left). Dot plots showing the proportion change of microglia among whole brain cells after PLX5622 treatment (right). n = 4 for each group; Student’s t test, two-sided (p = 0.0010) B) Dot plots showing the number of infiltrating immune cells among whole brain cells at 5 days post tMCAO. n = 3 for each group; Multiple t test [Macrophage (CD45^hi^CD11b^+^Ly6G^-^): p = 0.011937, B cells (CD45^hi^CD11b^-^CD3^-^CD19^+^): p = 0.718051, T cell (CD45^hi^CD11b^-^CD3^+^) = 0.844171, NK cell (CD45^hi^CD11b^-^CD3^-^CD19^+^) = 0.139847, Neutrophil (CD45^hi^CD11b^+^Ly6G^+^)= 0.476194 ] C) Violin plot illustrating the selected marker genes of each cluster of the whole mouse brains at d14. D) Stacked bar plot showing cell clusters harvested from the ipsilateral brain hemisphere at 14 dpi. n = 2 biological replicates for each group. E) Volcano plots depict the results of differential expression analysis in PLX group compared to CD group. Differentially expressed genes (DEGs, log fold change > 0.5 or < 0.5, Bonferroni adjusted p-value < 0.05) are colored (yellow for upregulated DEGs and blue for downregulated DEGs). F) Flow cytometry was performed to quantify the numbers of various immune cells in the brain. Gating strategy: Macrophages (CD45^hi^CD3^-^CD11b^+^Ly6G^-^), Neutrophil (CD45^hi^CD3^-^CD11b^+^Ly6G^+^), NK cells (CD45^hi^CD3^-^CD11b^-^NK1.1^+^), B cells (CD45^hi^CD3^-^CD11b^-^CD19^+^).


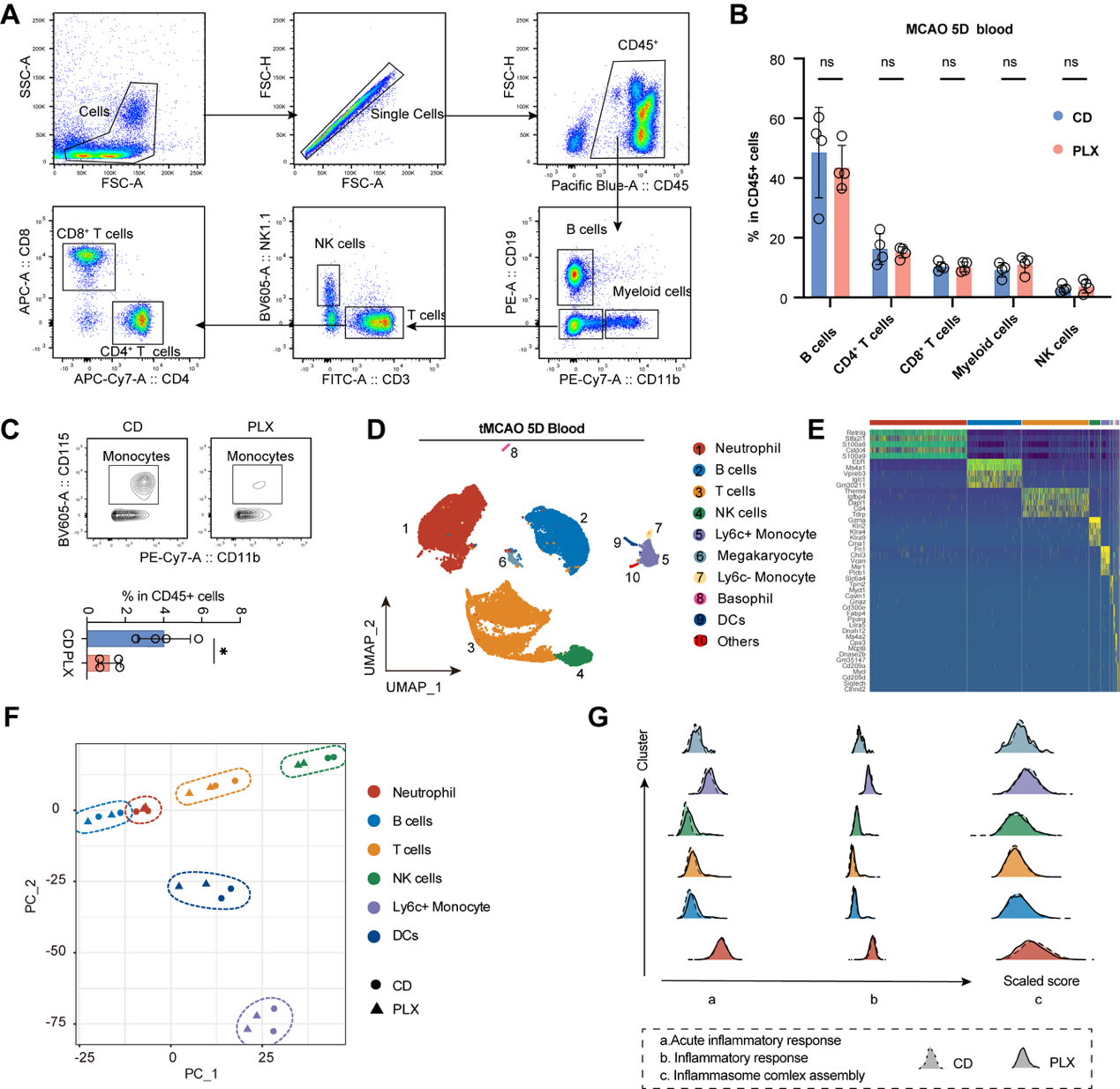


**Supplementary Figure 2.** A) Flow cytometry was performed to quantify the numbers of various immune cells in the blood at 5 days post tMCAO. Gating strategy: Myeloid (CD45^+^CD11b^+^), B cells (CD45^+^CD19^+^), NK cells (NK1.1^+^), CD4 T cells (CD45^+^CD11b^-^CD19^-^CD3^+^CD4^+^) and CD8 T cells (CD45^+^CD11b^-^CD19^-^CD3^+^CD8^+^). B) Dot plots showing the proportion of peripheral blood immune cells among CD45+ cells at 5 days post tMCAO. n=4 for each group. ns = not significant. Multiple t test (B cells: p = 0.966143, CD4 T cells: p = 0.966143, CD8 T cells: p = 0.966143, Myeloid cells: p = 0.896983, NK cells: p = 0.966143, Neutrophil: p = 0.896983) C) Flow cytometry was performed to quantify the proportion of monocytes in the blood at 5 days post tMCAO. Monocyte (CD45^+^CD11b^+^Gr-1^-^CD115^+^). n = 4 for each group. Welch’s t test. p = 0.0183 D) UMAP projection plot showing ten distinct cell types revealed by unsupervised clustering analysis in blood at 5 days post tMCAO. E) Heatmap showing the expression levels of top five marker genes in each cell type. F) PCA plot showing the distances of major immune cell types between the PLX group and CD group. G) Ridgeline plot summarizing AddModuleScore of inflammatory response functions of major immune cell types in blood at 5 days post tMCAO.


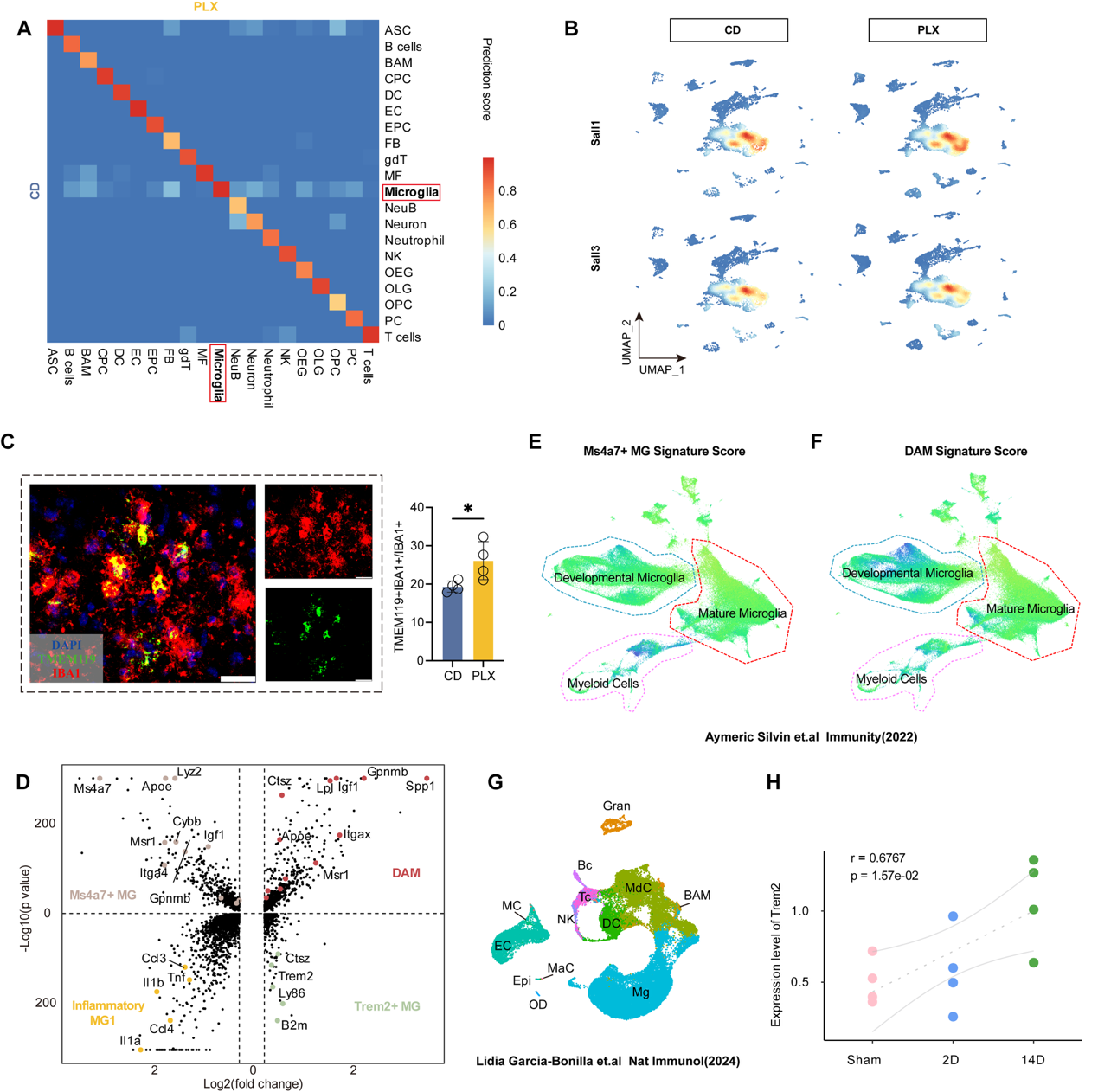


**Supplementary Figure 3:** A) Mapping of PLX group onto CD group clusters using canonical correlation analysis for MCAO 14dpi dataset. Prediction score is averaged across cells within a cluster. B) Density plots in the UMAP space showing the expression level of Sall1 and Sall3 from scRNA-seq of mouse brains at 14 dpi. C) Representative image showing the colocalization of TMEM119 (green) and IBA1 (red). Quantiﬁcation of TMEM119^+^IBA1^+^ proportion in IBA1^+^ cells. Scale bars, 20 µm. n = 4 for each group. Student’s t test, two-sided, p = 0.0015. D) Volcano plot showing upregulated genes across different subpopulations of microglia at 14 days post tMCAO. E-F) Signature heatmap of DEGs for Ms4a7+ MG and DAM across all M-Verse datasets (Amyeric Silvin’s dataset, https://macroverse. gustaveroussy.fr/2021_M-VERSE). G) UMAP projection plot showing cell clusters from the sham mice and the ipsilateral brain at 2, 14 dpi BAM–Border-associated Mø, Bc–B cells, DC–dendritic cells, EC–endothelial cells, Epi–epithelia-like cells, Eos.Bas–Eosinophil.Basophils, Gran–granulocytes, MaC–mast cells, MC–mural cells, Mg–microglia, MDMø–monocyte derived macrophages, Mo–monocytes, Neu–Neutrophil, NK–natural killer cells, Tc–T cells, OD–oligodendrocytes, pre–hematopoietic precursors, UC–unclassified. H) Dot plot showing the Two-tailed Pearson correlations of the expression level of Trem2 with times. n = 4/group.


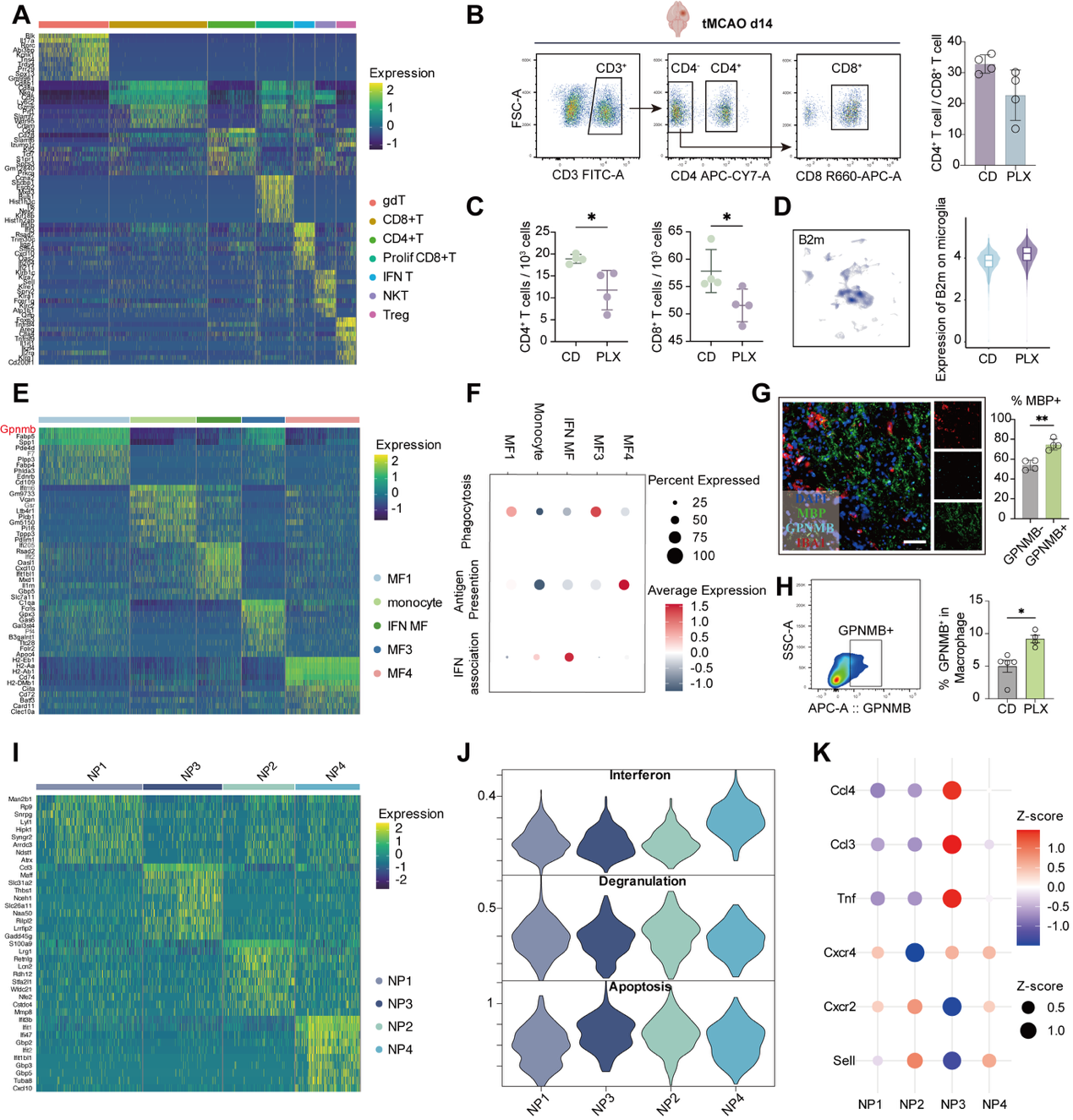


**Supplementary Figure 4:** A) Heatmap shows the 10 genes that are most highly expressed by each subpopulation of T cells. B) Flow cytometry was performed to quantify the numbers of CD4^+^ T cells and CD8^+^ T cells in the brain. Gating strategy: CD4^+^ T cells (CD3^+^ CD4^+^), CD8^+^ T cells (CD3^+^CD4^-^CD8^+^) (left). Dot plot showing the CD4^+^/CD8^+^ T cell ratio across different group (right). Student’s t test, two-sided. p = 0.0585 C) Dot plots showing the number of CD4^+^ T cells, CD8^+^ T cells (among 1000 cells in ipsilateral brain) with or without PLX5622 administration. n = 4 for each group. * p < 0.05. Student’s t test, two-sided (CD4+ T cells: p = 0.0212, CD8+ T cells: p = 0.0286). D) Density plots in the UMAP space showing the expression level of B2m from scRNA-seq of mouse brains at 14 dpi (left). Violin plot showing the expression levels of B2m in microglia across each group (right). E) Heatmap shows the 10 genes that are most highly expressed by each subpopulation of MF. F) Dot plot illustrating the module score of functional gene sets in MF across different subclsuters. G) Representative image showing the phagocytosis of MBP (green) by IBA1+ (red) GPNMB+ (cyan) cells. Scale bars, 50 µm. (left). Quantiﬁcation of MBP^+^ proportion in IBA1^+^GPNMB^+^ cells and IBA1^+^GPNMB^−^cells. n = 4 for each group. Student’s t test, two-sided, p = 0.0015. H) Flow cytometry was performed to quantify the proportion of macrophage subpopulations in brain post tMCAO. GMPNB^+^ Macrophage (CD45^hi^CD11b^+^Ly6G^-^GPNMB^+^) was exhibited (left). Dot plot showing the proportion change of GPNMB^+^ macrophage after early-phase PLX5622 treatment (right). Mann-Whitney test, two-sided, p = 0.0159 I) Heatmap shows the 10 genes that are most highly expressed by each subpopulation of neutrophils. J) Violin plot displaying the scores of functional gene sets in neutrophils across different groups. K) Dot plot showing the expression levels of inflammation associated genes in different neutrophil subpopulations.


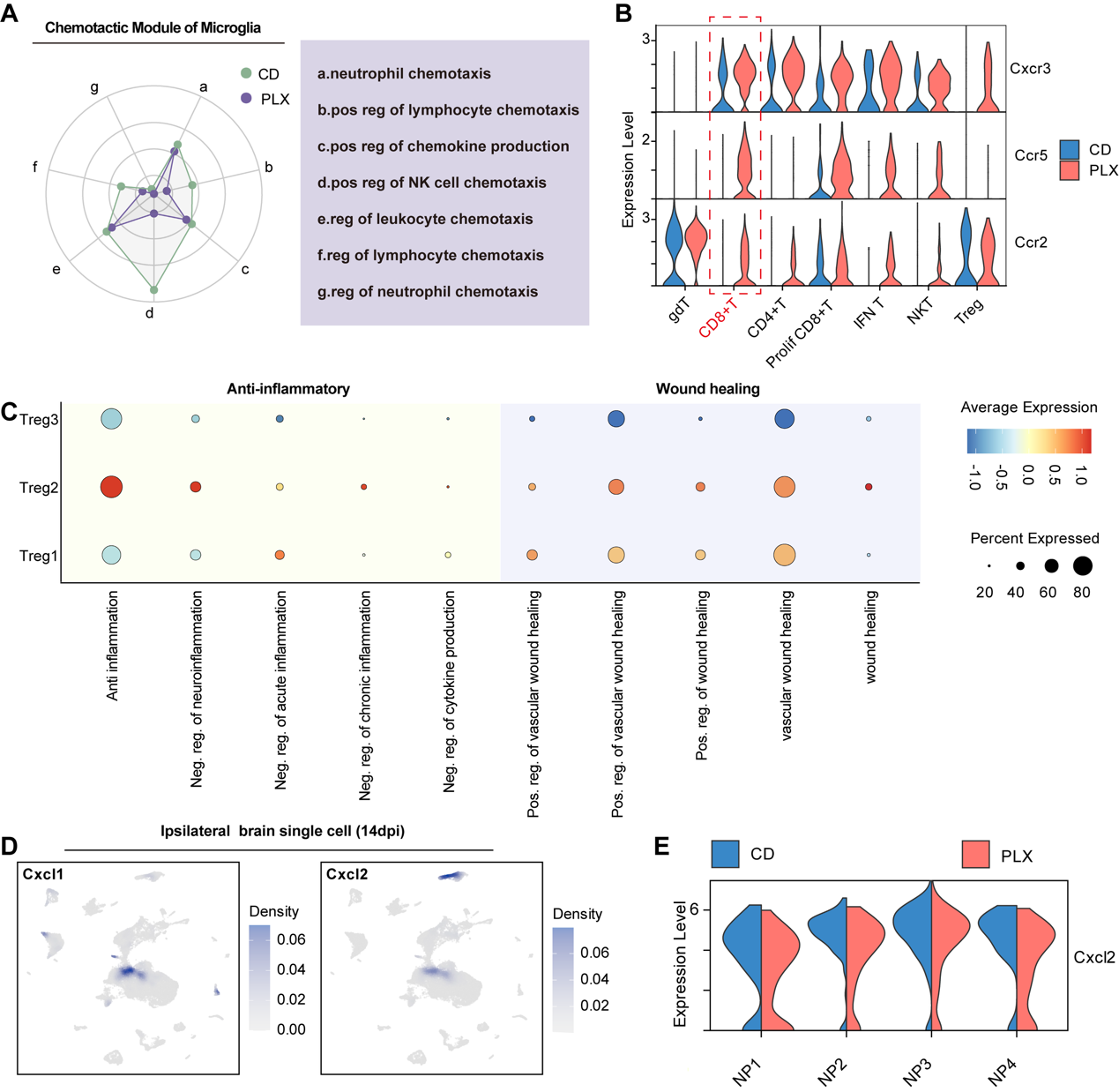


**Supplementary Figure 5:** A) Radar plot illustrating the module scores of chemokine-associated gene sets in microglia between the PLX5622 and control diet (CD) groups. B) Violin plot comparing the expression levels of Cxcr3, Ccr5, Ccr2 across T cell subtypes between the CD (red) and PLX (blue) groups. C) Dot plot illustrating the Gene Ontology (GO) scores of Tregs for anti-inflammatory and wound healing associated functions. D) Density plots in the UMAP space showing the expression level of Cxcl1 and Cxcl2 from scRNA-seq of mouse brains at 14 dpi. Scale bar represents densities based on Kerenel density estimation of gene expression. E) Violin plot comparing the expression levels of Cxcl2 across neutrophil subpopulations (NP1–NP4) between the CD (red) and PLX (blue) groups.


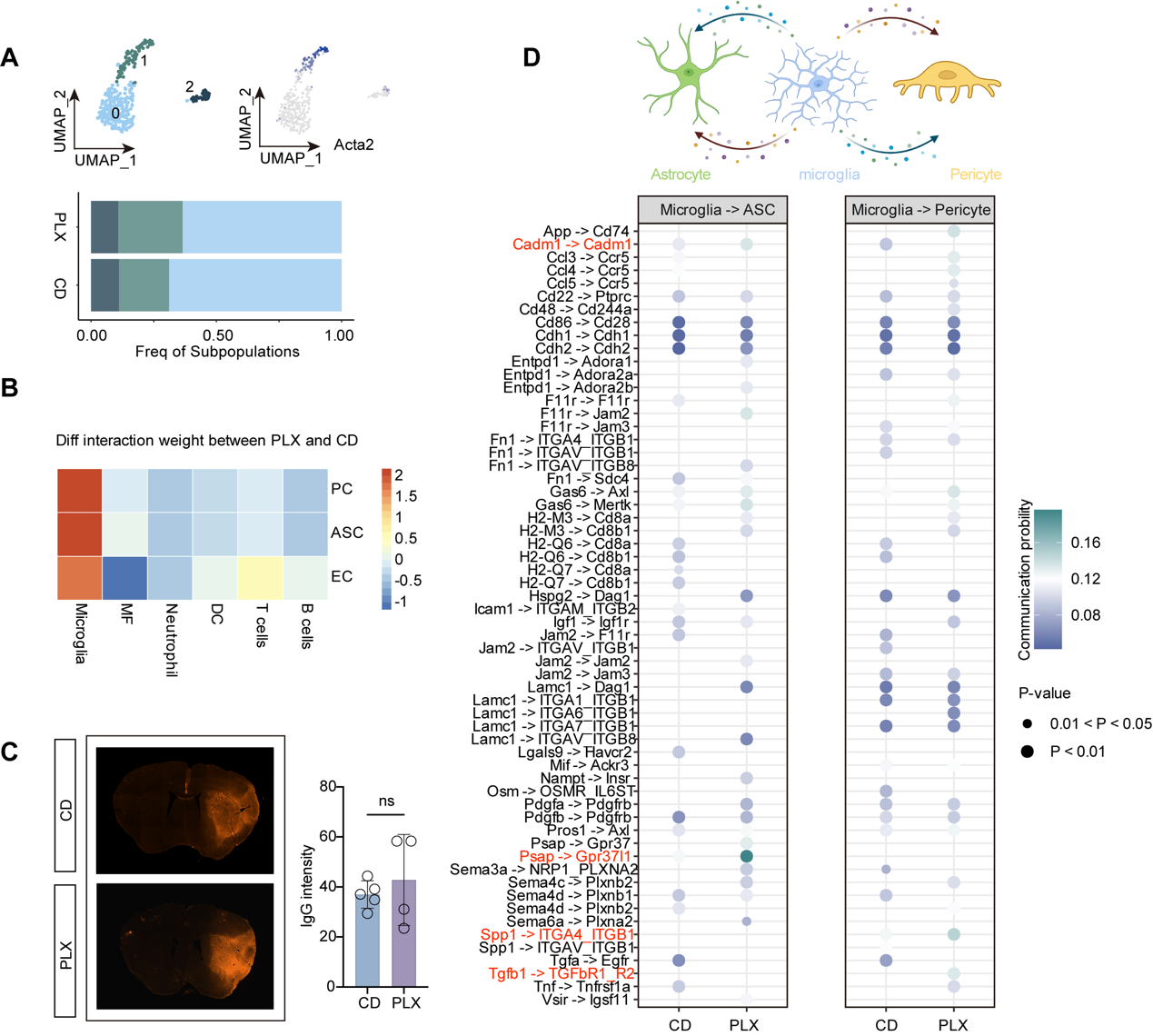


**Supplementary Figure 6:** A) UMAP projection of PC (upper left). UMAP projection illustrating the expression pattern of Acta2 across PC. Color represents the expression level of Acta2 (upper right). Proportion of pericyte subpopulations (lower). B) Heatmap showing the difference in interaction weight between the PLX group and CD group. C) Representative coronal sections immunolabeled for extravasated IgG (left) at 3 days post tMCAO, quantitative analysis of IgG intensity (right), n = 5 for CD group, n = 4 for PLX group. Student’s t test, two-sided (p = 0.5147). D) Dot plot showing the ligand-receptor interaction analysis between microglia and ASC or PC in CD and PLX groups. The color intensity indicating interaction strength and dot size reflecting statistical significance.


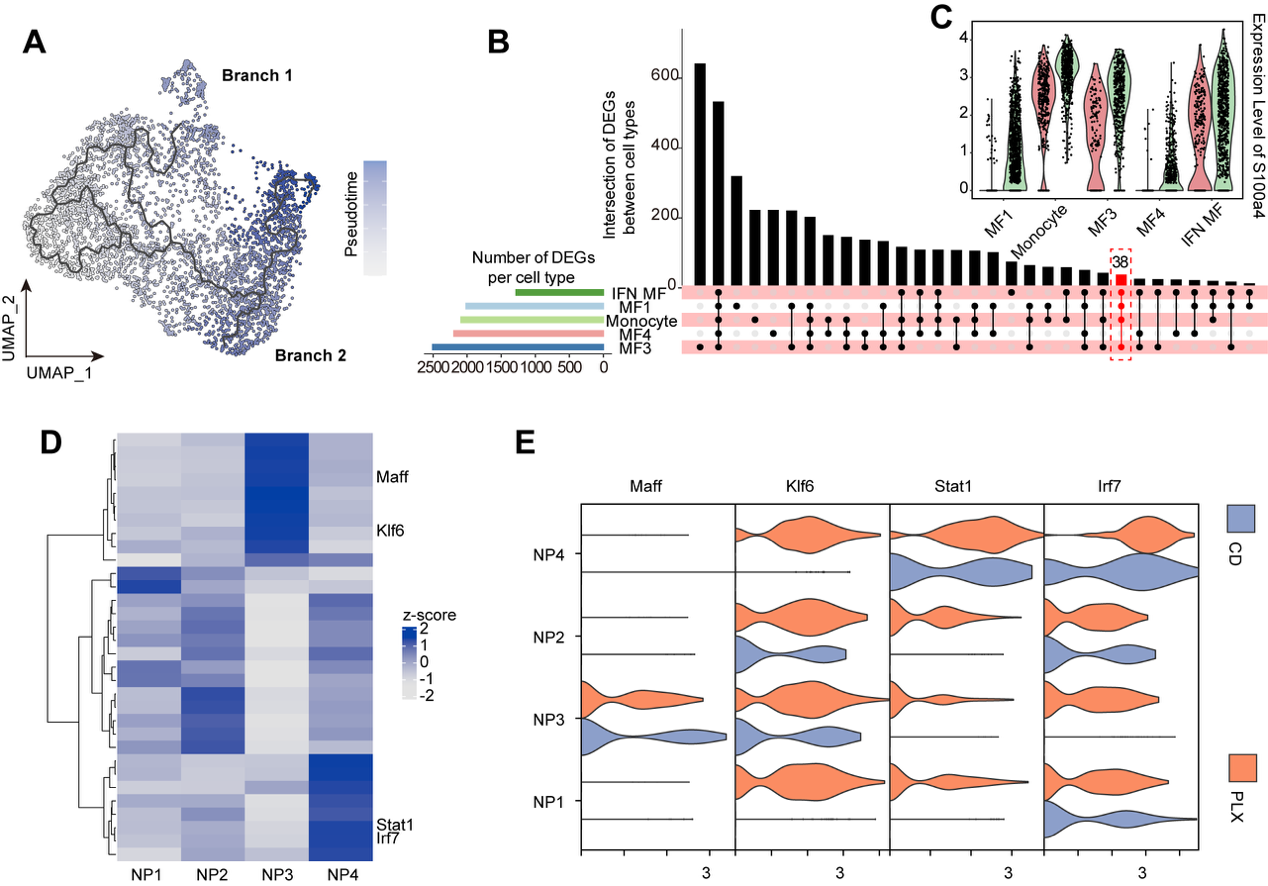


**Supplementary Figure 7:** A) Pseudotime trajectory of MF with monocle3. B) UpSet plot showing the intersection of differentially expressed genes (DEGs) among various MF subpopulations. Highlighted red box indicates a subset of genes shared among IFN MF, MF1, MF3 and monocyte. C) Violin plot illustrating the expression levels of S100a4 across different MF subpopulations between CD and PLX group. D) Heatmap showing the average regulon activities of representative TFs in each MF subpopulations derived from pySCENIC. The color gradient from light blue to deep blue indicates the relative expression levels from low to high. E) Violin plots comparing the expression levels of Irf7, Stat1, Klf6, and Maff across neutrophil subpopulations (NP1–NP4) between the CD (blue) and PLX (orange) groups.
